# Chiral manganese halide isomers: decoding the spatial stacking effect on second-harmonic generation circular dichroism

**DOI:** 10.1039/d5sc09866a

**Published:** 2026-03-10

**Authors:** Jing Li, Jianwu Wei, Qiulian Luo, Wei Pang, Hongming Liu, Peican Chen, Liya Zhou, Jin Zhong Zhang, Binbin Luo, Qi Pang

**Affiliations:** a School of Chemistry and Chemical Engineering, State Key Laboratory of Featured Metal Materials and Life-cycle Safety for Composite Structures, Guangxi Key Laboratory of Electrochemical Energy Materials, Guangxi University Nanning 530004 Guangxi P. R. China bbluo@gxu.edu.cn qipang@gxu.edu.cn; b Department of Chemistry and Biochemistry, University of California Santa Cruz California 95064 USA zhang@ucsc.edu

## Abstract

Chiral hybrid metal halides (CHMHs) represent promising candidates for chiral optoelectronics and nonlinear optics (NLO). However, the effect of spatial stacking on second-harmonic generation circular dichroism (SHG-CD) in CHMHs has not been well understood. Herein, we constructed two pairs of chiral manganese(ii) halide isomers (*R*)-α-Mn, (*S*)-α-Mn, (*R*)-β-Mn and (*S*)-β-Mn, which crystallize in chiral space groups *C*222_1_ and *P*2_1_, respectively. They exhibit near-unity photoluminescence quantum yields and efficient circularly polarized luminescence with an asymmetry factor (*g*_lum_) of ∼1.0 × 10^−3^. Additionally, these isomers show significant NLO responses with SHG intensities of 2.03 and 1.30 times that of KH_2_PO_4_ and polarization ratios of up to 0.87 and 0.57 for (*R*)-α-Mn and (*R*)-β-Mn, respectively. More importantly, (*R*)-α-Mn demonstrates an intense SHG-CD response with a SHG-CD factor (*g*_SHG-CD_) value of −0.56, about 1.86 times larger than that of (*R*)-β-Mn (−0.30). Compared to (*R*)- and (*S*)-β-Mn, the stacking mode of (*R*)- and (*S*)-α-Mn generates a more dense asymmetric hydrogen-bonding network, which greatly distorts [MnBr_4_]^2−^ tetrahedra and enhances the dipole moment, thereby significantly improving the SHG-CD value. This work elucidates the pivotal role of spatial stacking in chiral NLO materials.

## Abstract 2

The denser asymmetric hydrogen-bonding network of chiral (*R*)-α-Mn isomer greatly distorts [MnBr_4_]^2−^ tetrahedra, resulting in a superior SHG-CD response compared to that of (*R*)-β-Mn isomer.

## Graphical abstract

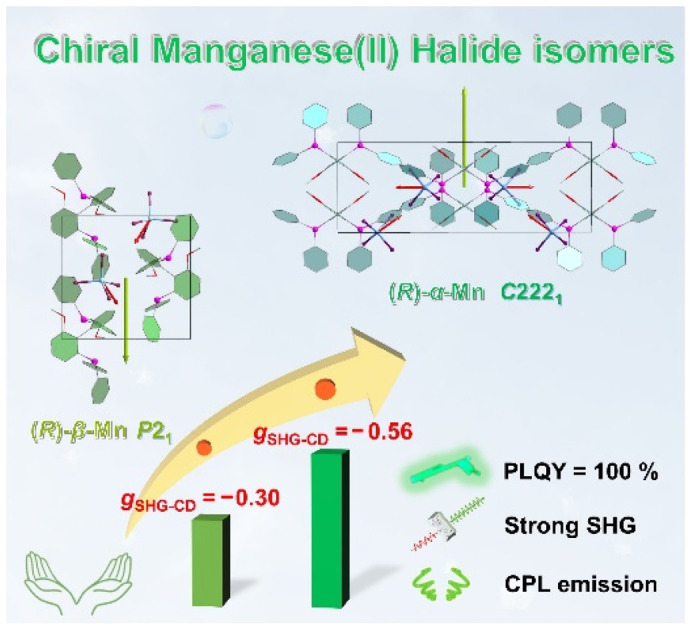

## Introduction

As a fundamental second-order nonlinear optical (NLO) process, second-harmonic generation (SHG) plays a pivotal role in ultrafast laser systems, optical sensing, and holographic imaging technologies.^1,2^ Conventional inorganic SHG materials exemplified by LiNbO_3_ and KH_2_PO_4_ (KDP) still suffer from challenges including synthetic complexity, limited wavelength response range, and weak SHG intensity.^3,4^

Chiral hybrid metal halides (CHMHs) have recently attracted enormous attention for their chiroptical and NLO properties due to their rich compositional and structural tunability.^5–9^ The second harmonic generation circular dichroism (SHG-CD) factor was proposed to evaluate the differential SHG response of chiral crystals to left circularly polarized (LCP) and right circularly polarized (RCP) light. The first SHG-CD of CHMHs with an anisotropy factor *g*_SHG-CD_ value of 0.62 was reported for (*R*-PEA)_1.5_PbBr_3.5_(DMSO)_0.5_ nanowires.^10^ Subsequently, various lead-free CHMHs were developed with *g*_SHG-CD_ values ranging from 0.26 to 0.98.^11–16^ In addition, manganese(ii)-based CHMHs also represent an emerging class of materials with significant potential in NLO and chiral photonics. Recent advances include the discovery of several chiral Mn-based NLO crystals, such as (*R*-MBA)MnCl_3_·CH_3_OH (0.40 × KDP), (*S*-MBA)_2_[MnCl_4_(H_2_O)_2_] (0.15 × KDP), (*S*-MBA)_2_[Mn_2_Cl_6_(H_2_O)_4_] (0.04 × KDP), (*R*-2-mpip)MnCl_4_·2H_2_O (0.05 × quartz) and (*R*)-(18-crown-6@ClMBA)_2_MnBr_4_ (12.7 × quartz).^17–20^ These advances highlight the advantages of manganese(ii)-based CHMHs in circularly polarized light (CPL) and NLO materials.^21,22^ Therefore, understanding the relationships between the crystal structure and SHG properties is critical for exploring Mn-based CHMHs with excellent SHG performance.

In general, the SHG performance of CHMHs highly correlates with their composition, molecular structures, spatial stacking and electronic states.^23^ Isomers, which share identical chemical composition but exhibit distinct spatial stacking, provide an effective approach to understand the effect of compositional arrangement on optical properties. For instance, three zero-dimensional (0D) hybrid copper(i) iodide isomers, α-/β-/γ-(BuPh_3_P)_2_Cu_2_I_4_, achieve full-colour tuneable emission from blue to red through modulating the spatial organization of their components.^24^ The isomeric pair, α- and β-(EtPPh_3_)_2_SbCl_5_·MeCN, shows distinct orange and red emissions by varying the pyramidal distortion of [SbCl_5_]^2−^ units.^25^ Hybrid Mn-based isomers, (C_19_H_18_P)_2_MnBr_4_, exhibit either piezoelectric or non-piezoelectric phases depending on their structural stacking, yet only the piezoelectric phase demonstrates self-recovering elastic mechanical luminescence.^26^ These findings demonstrate that subtle variations of spatial stacking can induce significant differences in optoelectronic properties even with identical compositions.^27,28^ Therefore, isomers serve as an ideal platform for understanding the effect of structural stacking modes on the SHG-CD factor. However, the role of molecular spatial stacking configurations in modulating SHG-CD signals of chiral isomers remains unclear.

In this study, two types of chiral Mn-based halide isomers, (*R*)-α-Mn, (*S*)-α-Mn, (*R*)-β-Mn and (*S*)-β-Mn were successfully synthesized through temperature-controlled crystallization. Single-crystal structure analysis indicates that (*R*)-α-Mn and (*S*)-α-Mn crystallize in chiral space group *C*222_1_, while (*R*)-β-Mn and (*S*)-β-Mn adopt chiral space group *P*2_1_. Both materials show intense green luminescence with near-unity photoluminescence quantum yields (PLQYs) and pronounced chiroptical properties, including a significant CD signal, CPL emission and SHG responses. More importantly, these two isomers show notable differences in SHG-CD responses, resulting from the varying degrees of tetrahedral distortion of [MnBr_4_]^2−^ units and differences in dipole moment magnitudes caused by their distinct compositional arrangement. This work provides new insights into the effect of chiral isomeric structures on SHG-CD properties and broadens the design strategies for multifunctional CHMH materials.

## Results and discussion

### Synthesis and structural characterization

**Scheme 1:**
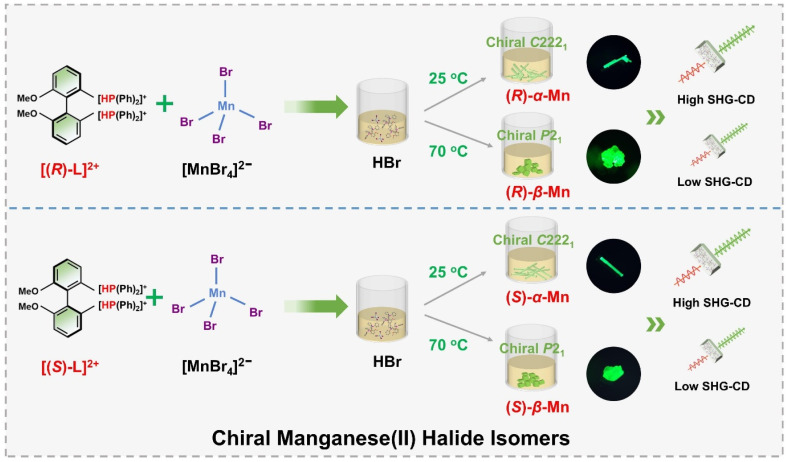
Schematic illustration of preparing the chiral hybrid manganese(ii) halide isomers.

Scheme 1 illustrates a temperature-controlled crystallization strategy for the preparation of two pairs of Mn-based CHMHs. Specifically, these isomers were obtained from a HBr solution of chiral ligand (*R*)- or (*S*)-6,6-dimethoxy-[1,1′-biphenyl-2,2′-diyl bis(diphenyl phosphine)] (denoted as (*R*)-l or (*S*)-l) and MnBr_2_·4H_2_O cooled at different temperatures. (*R*)-α-Mn and (*S*)-α-Mn single crystals were obtained at 25 °C, whereas (*R*)-β-Mn and (*S*)-β-Mn crystallized at 70 °C (see synthesis details in the SI). Single-crystal X-ray diffraction (XRD) indicates that (*R*)-α-Mn and (*S*)-α-Mn crystallize in orthorhombic space group *C*222_1_, whereas (*R*)-β-Mn and (*S*)-β-Mn adopt monoclinic space group *P*2_1_. Detailed crystallographic parameters are provided in Tables S1 and S2. The chiral (*R*)- and (*S*)-enantiomers show comparable unit cell parameters with an identical molecular formula of C_38_H_34_O_2_P_2_MnBr_4_. Fig. S1 shows the powder-XRD patterns of (*R*)-α-Mn, (*S*)-α-Mn, (*R*)-β-Mn and (*S*)-β-Mn, which match well with their corresponding simulation, confirming their high purity and crystallinity. Fig. S2 and S3 show the scanning electron microscopy (SEM) images of (*R*)-α-Mn and (*R*)-β-Mn crystals. (*R*)-α-Mn shows a rod-shaped morphology, while (*R*)-β-Mn displays a bulk-like shape. The corresponding energy-dispersive spectroscopy (EDS) mapping further confirms the homogeneous distribution of elements in these crystals.

**Fig. 1:**
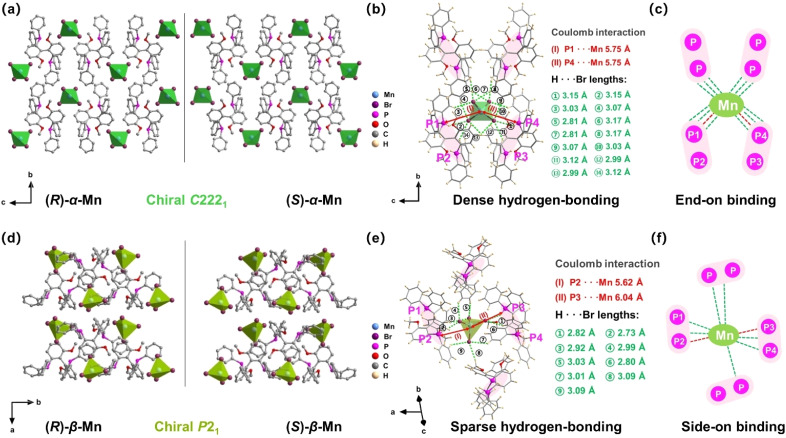
(a) Crystal structures of (*R*)-α-Mn and (*S*)-α-Mn along the *a*-axis. (b) Hydrogen-bonding interactions around the [MnBr_4_]^2−^ tetrahedron in (*R*)-α-Mn. (c) Schematic diagram of the hydrogen bonding interactions in (*R*)-α-Mn. (d) Crystal structures of (*R*)-β-Mn and (*S*)-β-Mn along the *c*-axis. (e) Hydrogen-bonding interactions around the [MnBr_4_]^2−^ tetrahedron in (*R*)-β-Mn. (f) Schematic diagram of the hydrogen bonding interactions in (*R*)-β-Mn. Red and green dashed lines represent P–H⋯Br and C–H⋯Br bonds, respectively.

The structures of these chiral crystals are shown in Fig. 1. Both enantiomer pairs (*R*)-α-Mn, (*S*)-α-Mn, (*R*)-β-Mn and (*S*)-β-Mn display mirror-image geometries (Fig. 1a and d). These crystals possess a unique 0D structure with isolated [MnBr_4_]^2−^ tetrahedra spatially separated and surrounded by chiral organic cations. The shortest Mn–Mn distances (Fig. S4) are 8.87 Å for (*R*)-α-Mn and (*S*)-α-Mn, which are smaller than those of (*R*)-β-Mn and (*S*)-β-Mn (9.35 Å). The component packing is predominantly driven by strong Coulomb interactions between the chiral cations and [MnBr_4_]^2−^ anions and is further stabilized by an extensive hydrogen-bonding network involving intermolecular C–H⋯Br and P–H⋯Br interactions. Fig. 1b and e show the stacking modes of chiral organic molecules and [MnBr_4_]^2−^ tetrahedra. In (*R*)-α-Mn and (*S*)-α-Mn, the shortest distance between the Mn atom and the P atoms of two adjacent cations is 5.75 Å, whereas the distances are 5.62 Å (P2–Mn) and 6.04 Å (P3–Mn) in (*R*)-β-Mn and (*S*)-β-Mn.

For clarity and ease of understanding, we define the spatial stacking mode as “end-on binding” for (*R*)-α-Mn and (*S*)-α-Mn, and as “side-on binding” for (*R*)-β-Mn and (*S*)-β-Mn (Fig. 1c and f). These crystals exhibit distinctly different hydrogen-bonding networks. Hydrogen bonds are exclusively considered for analysis when the H⋯X bond distances fall within the van der Waals limit (≤3.18 Å), as interactions beyond this distance are significantly weakened.^29,30^ In (*R*)-α-Mn and (*S*)-α-Mn, the stacking mode facilitates more dense asymmetric hydrogen-bonding networks around the [MnBr_4_]^2−^ tetrahedra, wherein multiple chiral organic cations cooperatively interact with [MnBr_4_]^2−^. In contrast, the [MnBr_4_]^2−^ tetrahedron is nearly entirely encapsulated by two chiral organic cations in (*R*)-β-Mn and (*S*)-β-Mn, which sterically hinder interactions with other surrounding chiral species, resulting in a sparse hydrogen-bonding network. This distinction may lead to a significant difference in their chiral expression. The structural distortions of these compounds were further evaluated using the following equations:^31,32^1
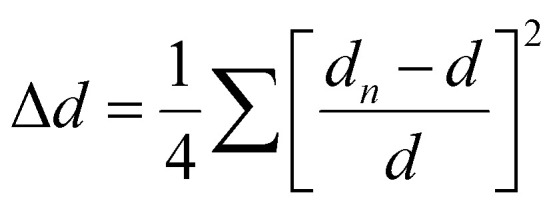
2
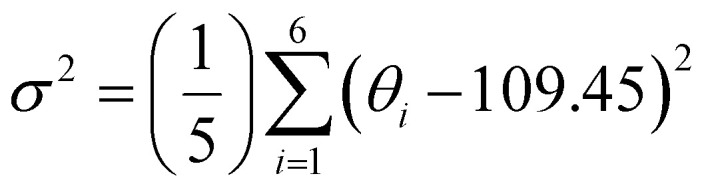


In eqn (1), Δ*d* represents the difference in bond lengths, where *d* and *d*_*n*_ denote the mean and individual Mn–Br bond lengths. In eqn (2), *θ*_*i*_ denotes the individual Br–Mn–Br angle and *σ*^2^ indicates the variance in tetrahedral bond angles. The Δ*d* values are calculated to be 3.46 × 10^−5^ and 5.39 × 10^−5^ for (*R*)-α-Mn and (*S*)-α-Mn, while 1.62 × 10^−5^ and 1.92 × 10^−5^ for (*R*)-β-Mn and (*S*)-β-Mn. The calculated *σ*^2^ values are 19.56 and 19.11 for (*R*)-α-Mn and (*S*)-α-Mn, while 28.92 and 28.28 for (*R*)-β-Mn and (*S*)-β-Mn. Unlike Δ*d*, which quantifies the deviation of Mn–Br bond lengths from their mean value, *σ*^2^ primarily characterizes the angular distortion of the tetrahedral geometry relative to its ideal configuration. The Δ*d* values of (*R*)-α-Mn and (*S*)-α-Mn are approximately double those of (*R*)-β-Mn and (*S*)-β-Mn, indicating a significantly pronounced degree of tetrahedral distortion in (*R*)-α-Mn and (*S*)-α-Mn.

The pronounced bond length distortion in (*R*)-α-Mn and (*S*)-α-Mn is attributed to the formation of a denser asymmetric hydrogen-bonding network, driven by their specific spatial stacking arrangement. Hirshfeld surface analysis of (*R*)-α-Mn and (*R*)-β-Mn crystals (Fig. S5) reveals dominant P–H⋯Br interactions between the [MnBr_4_]^2−^ tetrahedra and chiral organic cations, further corroborating the presence of robust asymmetric hydrogen-bonding within the lattice.^33–35^

### Photophysical properties

**Fig. 2:**
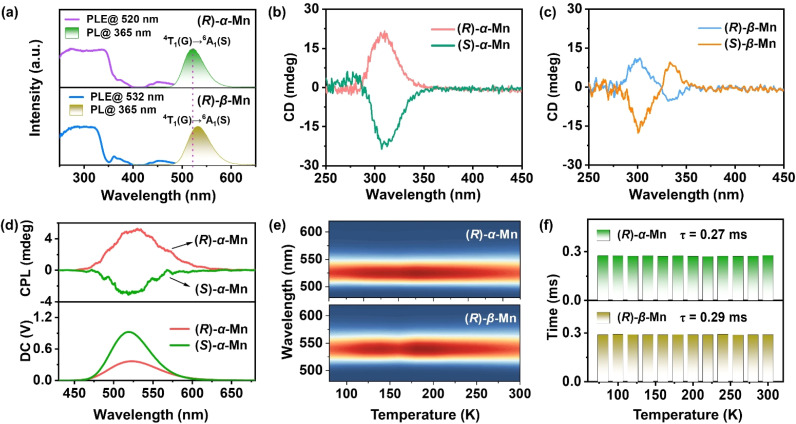
(a) PL excitation and PL spectra of (*R*)-α-Mn and (*R*)-β-Mn crystals. (b and c) CD signals of (*R*)-α-Mn, (*S*)-α-Mn, (*R*)-β-Mn and (*S*)-β-Mn samples. (d) CPL and DC spectra of (*R*)-α-Mn and (*S*)-α-Mn. (e) Temperature-dependent PL spectra of (*R*)-α-Mn and (*R*)-β-Mn crystals recorded from 80 to 300 K. (f) Fitting results of temperature-dependent PL decay for (*R*)-α-Mn and (*R*)-β-Mn.

Fig. S6 shows the UV-vis absorbance spectra of these chiral crystals. All (*R*)-α-Mn, (*S*)-α-Mn, (*R*)-β-Mn and (*S*)-β-Mn show characteristic bands (350–450 nm), corresponding to the d–d electronic transitions of Mn^2+^. Using the Kubelka–Munk function, the optical band gaps of (*R*)-α-Mn and (*R*)-β-Mn were calculated to be 2.20 eV and 2.10 eV, respectively.^36^Fig. 2a and S7 present the PL spectra of (*R*)-α-Mn, (*S*)-α-Mn, (*R*)-β-Mn and (*S*)-β-Mn. The excitation spectra of these crystals exhibit nearly identical energy profiles with two characteristic bands: ^6^A_1_ → ^4^D transition (330–390 nm) and ^6^A_1_ → ^4^G transition (430–500 nm), respectively. Under 365 nm excitation, the emission spectra of (*R*)-α-Mn and (*S*)-α-Mn exhibit a peak at 520 nm, whereas a peak centre at 532 nm is found for (*R*)-β-Mn and (*S*)-β-Mn. Fig. S8 shows the International Commission on Illumination (CIE) coordinates of (0.20, 0.70) and (0.26, 0.68) for (*R*)-α-Mn and (*R*)-β-Mn, respectively. Both compounds display green luminescence, attributed to the ^4^T_1_(G) → ^6^A_1_(S) transition of tetrahedrally coordinated Mn^2+^ ions. The minor variation in the emission peak positions is due to similar crystal field environments (Fig. S9).^37^ Randomly selected crystals of (*R*)-α-Mn, (*S*)-α-Mn, (*R*)-β-Mn and (*S*)-β-Mn from different batches were subjected to PLQY testing (Fig. S10). Both crystals exhibit near-unity PLQY (∼100%), representing the highest values reported to date for chiral crystalline materials. Such remarkable PLQY is attributed to the effective separation of [MnBr_4_]^2−^ luminescent centres by bulky chiral cations, wherein the increased Mn–Mn distance suppresses non-radiative energy transfer pathways.^38^ Fig. S11 presents the PL decay curves of the samples, which all follow monoexponential decay kinetics. The measured luminescence lifetimes are ∼0.28 ms for (*R*)-α-Mn and ∼0.29 ms for (*R*)-β-Mn, agreeing well with the characteristic of the spin-forbidden d–d transitions of tetrahedrally coordinated Mn^2+^ ions.

Wavelength-dependent PL excitation and emission spectra further confirmed the single luminescent centre of Mn^2+^ in these crystals (Fig. S12).

### Chiral optical properties

To demonstrate the chiral properties of these Mn-based isomers, CD spectra of the crystals were measured in a KBr matrix. Linear birefringence and linear CD were eliminated by flipping thin sheets.^17,39,40^Fig. 2b and c show the CD spectra of (*R*)-α-Mn, (*S*)-α-Mn, (*R*)-β-Mn and (*S*)-β-Mn with significant mirror symmetry in the range of 290–350 nm. The observed CD peaks are consistent with the corresponding absorbance spectra (Fig. S13). The CD spectra of chiral organic salts are significantly different from those of Mn-based isomers, further indicating the successful transfer of chirality to the entire crystal (Fig. S14).^40^ The CD anisotropy factor (*g*_CD_) is calculated according to eqn (3).^41,42^

3 *g*_CD_ = CD/(32 980 × absorbance)

The calculated CD anisotropy factor *g*_CD_ values of (*R*)-α-Mn and (*S*)-α-Mn are 8.7 × 10^−4^ and −9.7 × 10^−4^, respectively, which are larger than those of (*R*)-β-Mn (2.0 × 10^−4^) and (*S*)-β-Mn (−3.6 × 10^−4^) (Fig. S15). Fig. 2d and S16 show the CPL and direct current (DC) spectra of (*R*)-α-Mn, (*S*)-α-Mn, (*R*)-β-Mn and (*S*)-β-Mn. These spectra exhibit good agreement with their corresponding emission peaks. The asymmetry of the CPL is quantified by the asymmetry factor (*g*_lum_),^43,44^ which is defined as follows:

4 *g*_lum_ = 2 × (*I*_L_ − *I*_R_)/(*I*_L_ + *I*_R_)

where *I*_L_ and *I*_R_ stand for the left- and right-CPL intensities, respectively. As shown in Fig. S17, the experimentally determined *g*_lum_ maxima values for (*R*)-α-Mn and (*S*)-α-Mn are +1.0 × 10^−3^ and −0.31 × 10^−3^, respectively, whereas the *g*_lum_ values for (*R*)-β-Mn and (*S*)-β-Mn are +0.18 × 10^−3^ and −0.28 × 10^−3^, which are comparable with other Mn-based halides (Table S3). Chiral transfer efficiency may correlate with the density of the hydrogen-bonding network.^45,46^ In (*R*)-α-Mn and (*S*)-α-Mn, a dense hydrogen-bonding network induces larger asymmetric distortion in [MnBr_4_]^2−^. This efficiently transmits chirality from the molecular component to the inorganic polyhedra. In contrast, the sparse hydrogen-bonding networks in (*R*)-β-Mn and (*S*)-β-Mn induce minimal structural distortion, leading to poor chiral transfer and small *g*_CD_ and *g*_lum_ responses.

### Temperature-dependent PL

Fig. 2e shows the PL spectra of (*R*)-α-Mn and (*R*)-β-Mn across the temperature range of 80 to 300 K. The luminescence peak positions and intensities of (*R*)-α-Mn and (*R*)-β-Mn exhibit negligible variation with increasing temperature, indicating that temperature-induced non-radiative transitions are effectively suppressed by hydrogen-bonding interactions in these crystals.^47^ Fig. S18 presents the temperature-dependent PL decay curves (80–300 K) of (*R*)-α-Mn and (*R*)-β-Mn. Both crystals demonstrate mono-exponential decay characteristics with fitted lifetimes of 0.27 ms for (*R*)-α-Mn and 0.29 ms for (*R*)-β-Mn (Fig. 2f). This temperature-independent decay behaviour also confirms the highly suppressed non-radiative relaxation processes within this temperature range, explaining the observed near-unity PLQY. Fig. S19 presents the temperature-dependent PL spectra of (*R*)-α-Mn and (*R*)-β-Mn (300–420 K). The (*R*)-α-Mn retains 81.9% of its initial PL intensity at 420 K, much higher than that of (*R*)-β-Mn (57.0%), demonstrating the exceptional thermal quenching resistance of (*R*)-α-Mn enabled by the strong hydrogen bonding interaction. Furthermore, the thermal activation energy (*E*_a_) for exciton dissociation can be estimated by applying the Arrhenius equation.5
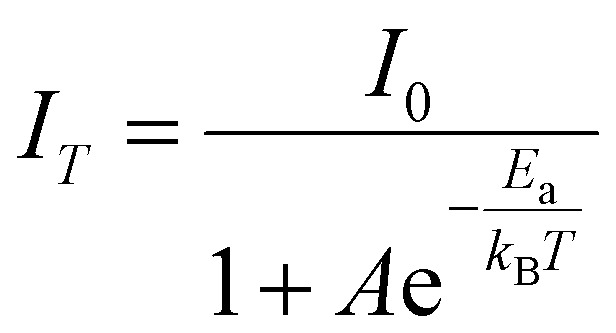
where *I*_0_ stands for the initial luminescence intensity and *k*_B_ is the Boltzmann constant. The results showed that the *E*_a_ values of (*R*)-α-Mn and (*R*)-β-Mn were 229 meV and 180 meV, respectively (Fig. S20). A higher thermal activation energy implies that these crystals exhibit high thermal stability at elevated temperatures. Fig. S21 presents the powder thermogravimetric analysis of (*R*)-α-Mn and (*R*)-β-Mn. No significant mass loss was observed below 180 °C, affirming their excellent thermal stability. This characteristic is crucial for ensuring the practical application of these materials in optoelectronic devices.

### Density functional theory calculations

**Fig. 3:**
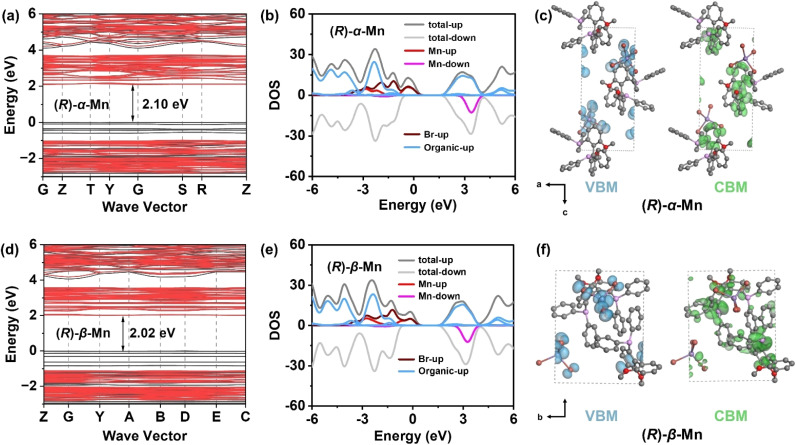
(a) Calculated band structure of (*R*)-α-Mn. (b) DOS of (*R*)-α-Mn. (c) Partial charge density plots for the VBM (left) and CBM (right) of (*R*)-α-Mn. (d) Calculated band structure of (*R*)-β-Mn. (e) DOS of (*R*)-β-Mn. (f) Partial charge density plots for the VBM (left) and CBM (right) of (*R*)-β-Mn.

To further understand the electronic structure and photophysical properties of these crystals, density functional theory (DFT) calculations were conducted to elucidate the electronic band structure and density of states (DOS). Fig. 3a and d show the electronic band structure of (*R*)-α-Mn and (*R*)-β-Mn. The band edge of these chiral crystals shows relatively flat dispersion, indicating the weak electronic coupling between [MnBr_4_]^2−^ clusters caused by the extended Mn–Mn distances. The calculated bandgap values are 2.10 and 2.02 eV for (*R*)-α-Mn and (*R*)-β-Mn, respectively, which are smaller than the experimental values. Such a difference is due to the underestimation of the Perdew–Burke–Ernzerhof (PBE) pseudopotential.^48^Fig. 3b and e show the DOS diagrams of (*R*)-α-Mn and (*R*)-β-Mn, respectively.

The valence band maximum (VBM) is primarily composed of Mn-3d and Br-4p orbitals, while the conduction band minimum (CBM) is dominated by the chiral organic molecule, consistent with the electron density distribution diagrams. Fig. 3c and f display the partial charge density maps corresponding to the VBM and CBM of the (*R*)-α-Mn and (*R*)-β-Mn crystals. The results indicate that the VBM and CBM of these chiral crystals are predominantly localized on discrete clusters and ligands, respectively, exhibiting significant spatial charge separation. This separation may suppress non-radiative electron–hole recombination, thereby enhancing the luminescence efficiency.^49,50^

### X-ray scintillation properties

**Fig. 4:**
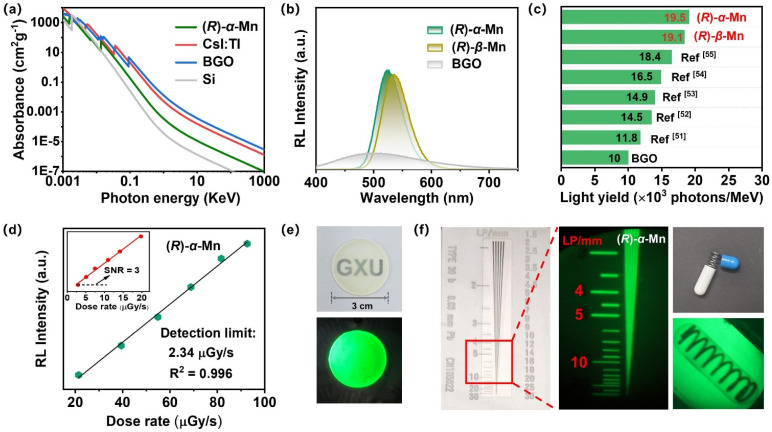
(a) Photoelectric absorbance coefficient of (*R*)-α-Mn, CsI:TI, BGO, and Si as a function of X-ray energy. (b) RL spectra of (*R*)-α-Mn, (*R*)-β-Mn and BGO. (c) Comparison of light yields in chiral manganese halide scintillators.^51–55^ (d) RL intensity as a function of the X-ray dose rate for (*R*)-α-Mn. (e) Fabricated scintillator film under natural light and UV light. (f) X-ray image showing the outline of a standard X-ray test specimen and the metal spring inside the capsule.

**Fig. 5:**
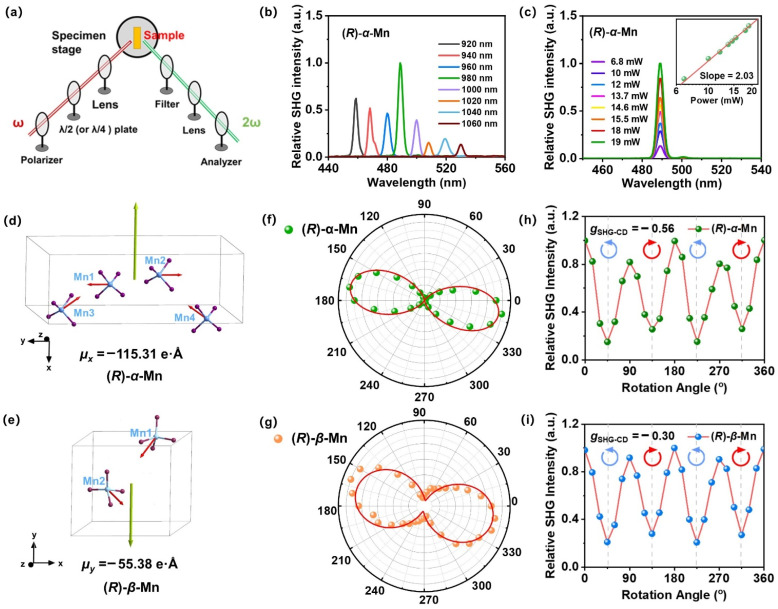
(a) Schematic diagram of the SHG system. (b) SHG spectra of (*R*)-α-Mn at various incident wavelengths. (c) Power-dependent SHG of the (*R*)-α-Mn crystal; the inset shows the logarithmic linear fit of the SHG intensity. (d and e) Calculated dipole moments of the [MnBr_4_]^2−^ central coordination units in (*R*)-α-Mn and (*R*)-β-Mn unit cells. (f and g) The polarization-dependent SHG response of (*R*)-α-Mn and (*R*)-β-Mn samples. (h and i) SHG intensity of (*R*)-α-Mn and (*R*)-β-Mn samples under LCP and RCP illumination.

We conducted a systematic investigation into their X-ray scintillation properties. Fig. 4a shows X-ray absorbance spectra for (*R*)-α-Mn simulated based on the photon cross-section database. At the same photon energy, (*R*)-α-Mn demonstrates high X-ray absorption coefficients, suggesting that these crystals are promising candidates for X-ray scintillators. Fig. 4b shows the radioluminescence (RL) spectra of (*R*)-α-Mn, (*R*)-β-Mn and Bi_4_Ge_3_O_12_ (BGO) under X-ray irradiation, which are similar to their corresponding UV-excited PL spectra. The light yields of (*R*)-α-Mn and (*R*)-β-Mn are measured to be 19 579 and 19 173 photons per MeV, significantly exceeding those of previously chiral Mn-based halide materials (Fig. 4c). The (*R*)-α-Mn and (*R*)-β-Mn samples have limits of detection of 2.34 and 4.21 µGy_air_ s^−1^, respectively (Fig. 5d and S22). Both values meet the requirement of 5.5 µGy_air_ s^−1^ for X-ray diagnosis. To prepare a flexible scintillation screen, we mixed (*R*)-α-Mn and (*R*)-β-Mn powders with polymethyl methacrylate (PMMA) (Fig. 4e). The scintillator screen emits uniform green light under ultraviolet illumination and has a thickness of approximately 0.18 mm (Fig. S23). The spatial resolution of the (*R*)-α-Mn and (*R*)-β-Mn scintillator screens was evaluated at a dose rate of 55 µGy_air_ s^−1^. Using a standard line pair card (TYPE 39 b), the spatial resolution was determined to be 5.0 lp per mm (Fig. 4f and S24). In addition, imaging experiments on in-capsule springs further confirmed the excellent performance of these materials in planar X-ray imaging, highlighting the promising applications of these chiral Mn-based crystals as emerging chiral scintillators.

The luminescence output of X-ray-excited scintillators originates from the efficient exciton energy transfer to the luminescence centre. When the chemical composition, PL, and PLQY are similar, the scintillation light output will be highly comparable. Therefore, despite the different crystal stacking arrangements of (*R*)-α-Mn and (*R*)-β-Mn, these crystals exhibit comparable performance for X-ray scintillators.

### SHG properties

The non-centrosymmetric chiral structures of (*R*)-α-Mn, (*S*)-α-Mn, (*R*)-β-Mn and (*S*)-β-Mn crystals confer significant second-order NLO properties. Fig. 5a illustrates the NLO experimental setup comprising the laser, polarisation control, and signal detection for the SHG test. Fig. 5b displays the SHG response spectrum of (*R*)-α-Mn in the 920–1060 nm excitation range. The results indicate the broad-spectrum characteristics of the SHG response for (*R*)-α-Mn, with the signal reaching a maximum at 490 nm. Fig. 5c shows the power-dependent SHG spectra of the (*R*)-α-Mn sample pumped at 980 nm. The slope value of 2.08 matches well with the theoretical value of 2, corroborating the second-order NLO nature of the process. Fig. S25 compares the SHG intensities of similarly sized (*R*)-α-Mn, (*S*)-α-Mn, (*R*)-β-Mn, and (*S*)-β-Mn samples with those of KDP, yielding relative values of 2.03, 1.73, 1.30, and 0.82, respectively. Additionally, the SHG properties of these chiral manganese single crystals were investigated using the Kurtz–Perry method.^65^ Fig. S25c shows the SHG intensity of these samples as a function of the particle size, confirming that both materials exhibit phase-matching capability at the fundamental wavelength. Notably, (*R*)-α-Mn displays significantly stronger SHG intensity than (*R*)-β-Mn across all measured particle size ranges. Using point charge models and DFT calculations, we systematically analysed the dipole moment distribution characteristics of (*R*)-α-Mn and (*R*)-β-Mn and their effects on the SHG intensity (Fig. 5d and e). As for (*R*)-α-Mn, four [MnBr_4_]^2−^ units exhibit a unique spatial stacking. Due to the opposite orientations of Mn1 and Mn2 centres, their dipole moments counteract each other, while the dipole moments of Mn3 and Mn4 centres are aligned along the *x*-direction to produce a net dipole moment (*µ*_*x*_ = −115.31 e Å). In (*R*)-β-Mn, two [MnBr_4_]^2−^ units generate a net dipole moment (*µ*_*y*_ = −55.38 e Å) oriented along the *y*-direction. The detailed electronic dipole moment and ionic dipole moment distribution characteristics of these crystals are shown in Table S4. The intensity of the SHG response exhibits a significant positive correlation with the molecular dipole moment, consistent with previous studies.^56,57^

Fig. 5f, g and S26 show the polarization-dependent SHG of (*R*)-α-Mn and (*R*)-β-Mn crystals, measured by rotating a *λ*/2 waveplate. The SHG signals exhibit a strong dependence on the polarization angle. Their polarization distribution patterns are well-fitted by a cos^4^ *θ* function and demonstrate pronounced anisotropy. The SHG anisotropy is defined as follows:^58,59^6
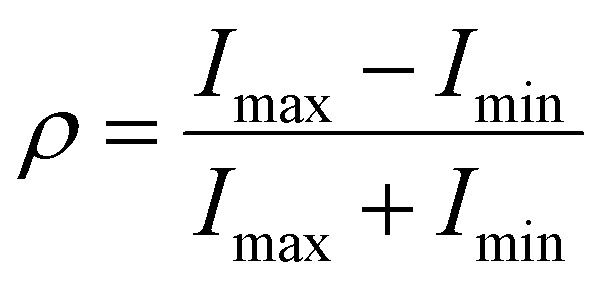
where *I*_max_ and *I*_min_ are the maximum and minimum values of the SHG intensity, respectively. We prepared three independent samples (*n* = 3) for both (*R*)-α-Mn and (*R*)-β-Mn and systematically evaluated their polarization-resolved SHG responses. These two isomers exhibited pronounced polarization anisotropy (Fig. S27) with good reproducibility. The average anisotropy parameter *ρ* is calculated to be 0.87 for (*R*)-α-Mn and 0.57 for (*R*)-β-Mn (Fig. S28).

### SHG-CD properties

We further investigated the SHG-CD properties of these Mn-based CHMHs. During the measurement, we replaced the *λ*/2 plate with a *λ*/4 plate to regulate the incident light from linearly polarized to LCP or RCP light. Fig. 5h and i show the SHG intensity of (*R*)-α-Mn and (*R*)-β-Mn at different rotation angles of the *λ*/4 plate. When the *λ*/4 plate rotates continuously from 0° to 360°, (*R*)-α-Mn and (*R*)-β-Mn single crystals exhibit significant differences in the SHG intensity between LCP and RCP. Subsequently, crystals of (*R*)-α-Mn, (*S*)-α-Mn, (*R*)-β-Mn, and (*S*)-β-Mn from different batches were randomly selected for circularly polarized nonlinear testing (Fig. S29). These chiral crystals exhibit distinct differences in the SHG intensity under LCP and RCP light illumination. The SHG signal of (*R*)-α-Mn under RCP light excitation was much larger than that under LCP light excitation, and the opposite trend was obtained for (*S*)-α-Mn. This phenomenon is also observed in (*R*)-β-Mn and (*S*)-β-Mn crystals. The chirality of NLO is evaluated through SHG-CD anisotropy,^60,61^ which is defined as follows:7
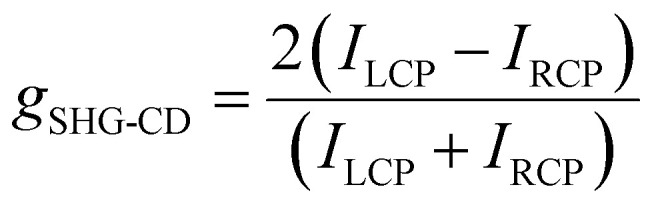
where *I*_LCP_ and *I*_RCP_ represent the SHG intensities under LCP and RCP light excitation, respectively. The calculated average *g*_SHG-CD_ values for (*R*)-α-Mn and (*S*)-α-Mn were −0.56 and 0.55, respectively, while those for (*R*)-β-Mn and (*S*)-β-Mn were −0.30 and 0.35, respectively (Fig. S30). These values are larger than those of other lead-free CHMHs (Table S5).

The significant anisotropy factor indicates that chirality has been effectively transferred to the [MnBr_4_]^2−^ tetrahedra, enabling their direct application in distinguishing LCP and RCP light. Based on the above crystallographic, chiroptical spectroscopic and SHG studies, we propose the following mechanism to elucidate the influence of spatial stacking on SHG-CD. In (*R*)-α-Mn, the spatial stacking mode enhances synergistic interactions among chiral cations, resulting in a dense asymmetric hydrogen-bonding network. This configuration facilitates efficient chiral transfer from the organic cations to the [MnBr_4_]^2−^ tetrahedra, thereby inducing more pronounced geometric distortion.^62–64^ Consequently, it is reasonable to conclude that the larger tetrahedral distortion in (*R*)-α-Mn will yield a stronger SHG-CD effect. This study provides important guidance for the design of high-performance SHG-CD materials.

## Conclusion

In summary, we have successfully synthesized two pairs of chiral manganese(ii) halide isomers, (*R*)-α-Mn, (*S*)-α-Mn, (*R*)-β-Mn and (*S*)-β-Mn, which serve as an ideal platform for deciphering the influence of spatial stacking on NLO properties. Both isomers exhibit near-unity PLQY and pronounced CPL activity, with the *g*_lum_ value reaching 1.0 × 10^−3^. Furthermore, they show strong SHG responses with intensities of 2.03 × KDP and 1.30 × KDP for (*R*)-α-Mn and (*R*)-β-Mn, respectively, along with high polarization ratios of 0.87 and 0.58. More importantly, the SHG-CD effect of (*R*)-α-Mn (*g*_SHG-CD_ = −0.56) is significantly stronger than that of (*R*)-β-Mn (*g*_SHG-CD_ = −0.30). This is attributed to the generation of a denser asymmetric hydrogen-bonding network in (*R*)- and (*S*)-α-Mn, which greatly distorts the [MnBr_4_]^2−^ tetrahedra and enhances their dipole moment. These isomers also demonstrate outstanding X-ray scintillation capabilities, making them promising candidates for chiral photonic and radiation detection applications. This study advances the understanding of the spatial stacking of chiral hybrid materials on regulating SHG-CD, and opens new avenues for designing multifunctional NLO systems with tailored performance.

## Author contributions

Q. Pang and B. Luo designed and directed the project. J. Li, J. Wei, Q. Luo and W. Pang performed the experiments and analysed the data. P. Chen, H. Liu, L. Zhou, and J. Zhang wrote the manuscript with input from all other authors.

## Conflicts of interest

There are no conflicts to declare.

## Supplementary Material

SC-OLF-D5SC09866A-s001

SC-OLF-D5SC09866A-s002

## Data Availability

The data supporting this article have been included as part of the supplementary information (SI). Supplementary information is available. See DOI: https://doi.org/10.1039/d5sc09866a. CCDC 2450541 for (*R*)-α-Mn, 2450542 for (*S*)-α-Mn, 2450543 for (*R*)-β-Mn, and 2450544 for (*S*)-β-Mn contain the supplementary crystallographic data for this paper.^66*a*–*d*^
