# Supplementary material for: Chiral manganese halide isomers: decoding the spatial stacking effect on second-harmonic generation circular dichroism
Source: Chem Sci. 2026 Mar 10;17(18):9061–71. doi: 10.1039/d5sc09866a (PMC12990912; doi:10.1039/d5sc09866a)
Supplement: SC-017-D5SC09866A-s001 [file SC-017-D5SC09866A-s001.pdf]

Supplementary Information (SI) for Chemical Science.
This journal is © The Royal Society of Chemistry 2026

Supporting Information

Chiral manganese halide isomers: decoding spatial stacking effect on second-harmonic generation circular dichroism

Jing Li[a], Jianwu Wei[a], Qiulian Luo[a], Wei Pang[a], Hongming Liu[a], Peican Chen[a], Liya Zhou[a], Jin Zhong Zhang[b], Binbin Luo*[a], and Qi Pang*[a]

[a]School of Chemistry and Chemical Engineering/State Key Laboratory of Featured Metal Materials and Life-cycle Safety for Composite Structures/ Guangxi Key Laboratory of Electrochemical Energy Materials, Guangxi University, Nanning 530004, Guangxi, P. R. China, *Corresponding authors: bbluo@gxu.edu.cn, qipang@gxu.edu.cn

[b]Department of Chemistry and Biochemistry, University of California, Santa Cruz, California 95064, United States, E-mail: zhang@ucsc.edu

Experimental Procedures

Material:

Manganese bromide tetrahydrate ($MnBr_2 \cdot 4H_2O$, 99.99%), (*R*)- or (*S*)- 6,6'-dimethoxy-[1,1'-biphenyl-2,2'-diyl bis(diphenylphosphine)], (*R*)-L and (*S*)-L, 99.9%, Polymethyl Methacrylate, Toluene (AR), Diethyl ether (AR), and Hydrobromic acid (HBr, 33 wt%) were purchased from Aladdin. These materials and chemicals were not subjected to additional purification before use.

Synthesis of (R)-α-Mn, (S)-α-Mn, (R)-α-Mn and (S)-β-Mn crystals:

The directed synthesis of these crystals was successfully achieved by a temperature-controlled solvent evaporation method. The specific preparation steps are as follows: In a 20 mL beaker, 0.20 mmol of the (*R*)-L or (*S*)-L ligand and 0.10 mmol of $MnBr_2 \cdot 4H_2O$ were sequentially added and dissolved in 2.0 mL of HBr solution. Subsequently, the solution was divided into two portions. One portion was incubated at room temperature for 5-7 days to obtain (*R*)-*α*-Mn or (*S*)-*α*-Mn single crystals, while the other portion was incubated on a heating table at 70 °C for 3-5 days to obtain (*R*)-*β*-Mn or (*S*)-*β*-Mn crystal products. The resulting crystals were washed three times with diethyl ether and then dried in a vacuum drying oven for 12 hours. The dried crystal samples were ground into fine powder for subsequent X-ray diffraction (XRD) and fluorescence spectroscopy analyses.

Characterizations:

Single-crystal X-ray diffraction (SC-XRD) data were collected on a Bruker D8 diffractometer equipped with MoKα radiation (λ = 0.71073 Å) operating at 50 kV and 30 mA. The chiral manganese(II) crystal structure was visually analyzed using OLEX2 and Diamond 3.2 software. Power X-ray diffraction (PXRD) data were collected at room temperature using a Bruker D8 Advance diffractometer equipped with Cu Kα (λ = 0.15405 nm) radiation (operating voltage: 40 kV, current: 30 mA). The scanning range was set to 5-50° with a scanning speed of 12°/min. Morphology and elemental analysis were performed using a field-emission scanning electron microscope (FE-SEM, Sigma 300) equipped with an energy-dispersive spectrometer (EDS). UV-Vis absorbance spectra of the samples were measured using a UV-2600 ultraviolet-visible spectrometer. Steady-state photoluminescence (PL) spectra, PL excitation spectrum, photoluminescence quantum yields (PLQY), and PL decay time were recorded using an FLS1000 fluorescence spectrometer at room temperature. Temperature-dependent PL spectra were acquired using an Oxford Optistat DN-V cryostat. Thermogravimetric analysis (TGA) was performed using a Mettler Toledo TGA2 thermogravimetric analyzer. Circular dichroism (CD) spectra were measured on a MOS-450 CD spectrometer and circularly polarised luminescence (CPL) spectra were recorded on JASCO CPL-300 solid-state spectrometer. CD testing was conducted by flipping the film, with the effects of linear birefringence and linear circular dichroism (LBLD) on the circular dichroism signal eliminated via the following formula: $CD_{true} = 0.5 \times (CD_{obs,front} + CD_{obs,back})$.[1] The scintillation characteristics were assessed utilizing a custom-built CuKα radiation X-ray imaging system, and imaging photographs were captured with a digital camera. The SHG testing system primarily consists of five components: laser light source, power and polarization control unit, focusing system, non-linear crystal, and a detection system. By employing a half-wave plate (λ/2) and a quarter-wave plate (λ/4), the polarization of the pump beam was modulated between linear and circular states. The reflected signal was collected from the front surface of the crystal. Under 980 nm laser excitation, power-dependent SHG spectra were acquired by progressively increasing the incident laser power.

Fabrication of X-ray scintillator thin films:

(*R*)-*α*-Mn and (*R*)-*β*-Mn crystals were ground into fine powder, and the powder was collected using a 400-mesh standard sieve. Subsequently, 2.5 mL of toluene containing 0.5 g of polymethyl methacrylate (PMMA) was stirred and heated at 60 °C for 2 hours to form a homogeneous solution. Then, 0.2 g of the sieved chiral manganese halide powder (*R*)-*α*-Mn or (*R*)-*β*-Mn was added, and the mixture was sonicated for 30 minutes to obtain a uniformly dispersed solution. Finally, the solution was poured into a circular mold with a diameter of 3 cm, left to stand for 20 minutes, and cured to obtain a transparent scintillating film. The thickness of the scintillator film was measured using a CQ-TOOLS thickness gauge.

DFT calculations

Density Functional Theory (DFT) calculations were performed using the Castep and Vasp software.[2, 3] The exchange-correlation interaction was treated within the generalized gradient approximation proposed by Perdew-Burke-Ernzerhof (GGA-PBE).[4] The geometric structures were optimized with a cutoff energy of 570 eV. During the structural relaxation, the convergence tolerances for energy, maximum force, maximum stress, and maximum displacement were set to 5.0×10^{-6} eV/atom, 0.01 eV/Å, 0.02 GPa, and 5.0×10^{-4} Å, respectively. Norm-conserving pseudopotentials were employed in this study. For relaxation purposes, the Brillouin zone was sampled using a K-point grid of 3 × 3 × 2. To account for strong electron correlations, the DFT+U approach was applied.[5, 6] Spin polarization method was adopted to describe the magnetic system.

Results and Discussion

Table S1 Crystal date and structure refinement for (*R*)-*α*-Mn and (*S*)-*α*-Mn crystals.

Identification code	(*R*)-*α*-Mn	(*S*)-*α*-Mn
Moiety formula	$C_{38}H_{34}O_2P_2MnBr_4$	$C_{38}H_{34}O_2P_2MnBr_4$
Formula weight	959.17	959.17
Temperature/K	295	301.44(12)
Crystal system	orthorhombic	orthorhombic
Space group	$C222_1$	$C222_1$
a/Å	8.870(6)	8.8222(4)
b/Å	24.954(17)	24.8809(10)
c/Å	18.197(13)	18.1166(7)
α/°	90	90
β/°	90	90
γ/°	90	90
Volume/Å^3	4028(5)	3976.7(3)
Z	4	4
ρ_{calc}g/cm^3	1.583	1.602
μ/mm^{-1}	4.406	4.462
F(000)	1892.0	1892.0
Crystal size/mm^3	0.23 × 0.23 × 0.2	0.23 × 0.23 × 0.2
Radiation	Mo Kα (λ = 0.71073)	Mo Kα (λ = 0.71073)
2θ range for data collection/°	3.958 to 49.976	4.496 to 52.734
Index ranges	-10 ≤ h ≤ 10, -29 ≤ k ≤29, -21 ≤ l ≤ 21	-11 ≤ h ≤ 10, -26 ≤ k ≤ 30, -20 ≤ l ≤ 22
Reflections collected	27857	8243
Independent reflections	3566 [R_{int} = 0.1471, R_{sigma} = 0.1241]	3794 [R_{int} = 0.0248, R_{sigma} = 0.0341]
Data/restraints/parameters	3566/195/195	3794/1/218
Goodness-of-fit on F^2	1.030	1.089
Final R indexes [I>=2σ (I)]	R_1 = 0.0647, wR_2 = 0.1564	R_1 = 0.0466, wR_2 = 0.1327
Final R indexes [all data]	R_1 = 0.1401, wR_2 = 0.1800	R_1 = 0.0574, wR_2 = 0.1379
Flack paeameter	-0.020(2)	0.007(16)
CCDC	2450541	2450542

Table S2 Crystal date and structure refinement for (*R*)-*β*-Mn and (*S*)-*β*-Mn crystals.

Identification code	(*R*)-*β*-Mn	(*S*)-*β*-Mn
Moiety formula	$C_{38}H_{34}O_2P_2MnBr_4$	$C_{38}H_{34}O_2P_2MnBr_4$
Formula weight	959.17	959.17
Temperature/K	303	301
Crystal system	monoclinic	monoclinic
Space group	$P2_1$	$P2_1$
a/Å	12.3302(11)	12.3199(6)
b/Å	11.2740(9)	11.2470(4)
c/Å	14.7720(14)	14.7379(6)
α/°	90	90
β/°	106.737(3)	106.670(2)
γ/°	90	90
Volume/Å^3	1966.5(3)	1956.29(14)
Z	2	2
ρ_{calc}g/cm^3	1.620	1.628
μ/mm^{-1}	4.512	4.535
F(000)	946.0	946.0
Crystal size/mm^3	0.2 × 0.2 × 0.2	0.2 × 0.2 × 0.2
Radiation	Mo Kα (λ = 0.71073)	Mo Kα (λ = 0.71073)
2θ range for data collection/°	4.62 to 50.7	4.63 to 56.614
Index ranges	$-14 \le h \le 14, -13 \le k \le 13, -17 \le l \le 17$	$-16 \le h \le 16, -13 \le k \le 15, -19 \le l \le 19$
Reflections collected	20323	22199
Independent reflections	7052 [R_{int} = 0.0667, R_{sigma} = 0.0916]	8865 [R_{int} = 0.0648, R_{sigma} = 0.0959]
Data/restraints/parameters	7052/1/422	8865/1/434
Goodness-of-fit on F^2	1.020	1.0492
Final R indexes [I>=2σ (I)]	R_1 = 0.0440, wR_2 = 0.0926	R_1 = 0.0470, wR_2 = 0.1016
Final R indexes [all data]	R_1 = 0.0720, wR_2 = 0.1025	R_1 = 0.0759, wR_2 = 0.1180
Flack paeameter	-0.015(9)	0.030(10)
CCDC	2450543	2450544

Table S3 The g_{lum}, PL peak position and PLQY values for known CPL-active Mn(II) complexes.

Chiral compounds	λ_{em} (nm)	PLQY	g_{lum} (10^{-3})	Ref
(*R*)-*α*-Mn	520	0.98	1.00	This work
(*R*)-*β*-Mn	532	0.98	0.21	This work
(*R*)-MPZMB	530	0.12	1.12	[7]
(*R*-2-mpip)$MnCl_4 \cdot 2H_2O$	660	0.13	1.20	[8]
(*R*)-[Me-Pr-DABCO]$MnBr_4$	525	0.87	48.0	[9]
(*R*)-XTDPO-$MnBr_2$	540	0.87	2.00	[10]
(*R*)-$C_{20}H_{28}Br_4MnN_2$	519	0.59	25.0	[11]
H_2(*R*-BDPP)]$MnBr_4$	523	1.00	2.00	[12]
(*R*)-Mn(Binapo)Br_2	629	0.21	5.10	[13]
(*R*)-$C_{24}H_{72}Cl_{10}Mn_3N_{12}$	638	0.45	7.10	[14]
(*R*-BrMBA)$_3MnBr_5$	634	0.42	0.90	[15]
(*R*)-[MBA-Me_3]$MnBr_4$	519	0.98	4.50	[16]
(*R*-3-quinuclidinol)$MnBr_3$	620	0.50	23.0	[17]
(*R*,*R*/*S*,*S*-DCDA)$Mn_{1-x}Zn_xCl_4$	528	0.86	7.80	[18]
(*R*-1-PPA)$_2MnBr_4$	530	0.09	10.0	[19]

Table S4 Calculated dipole moments and their ionic and electronic contributions for (*R*)-*α*-Mn and (*R*)-*β*-Mn crystals.

Sample	Contribution	x (e·Å)	y (e·Å)	z (e·Å)
(*R*)-*α*-Mn	lonic	-115.31	0.000	0.000
	Electronic	0.000	0.000	0.000
	Total	-115.31	0.000	0.000
(*R*)-*β*-Mn	lonic	0.000	-52.60	0.000
	Electronic	0.000	-2.780	0.000
	Total	0.000	-55.38	0.000

Table S5 The g_{SHG-CD} value comparison of these Mn-based isomers with previously reported chiral halides.

Chiral compounds	λ (nm)	g_{SHG-CD}	Ref
(*R*)-*α*-Mn	980	-0.56	This work
(*R*)-*β*-Mn	980	-0.30	This work
(*R*)-1-(1-NEA)$_2$CuCl$_4$	880	0.41	[20]
(*R*)-1-(2-NEA)$_2$CuCl$_4$	880	0.10	[20]
(*R*)-(3-aminopiperidine)PbI$_4$	1064	0.21	[21]
(*R*-MPEA)$_{1.5}$PbBr$_{3.5}$(DMSO)$_{0.5}$	850	0.62	[22]
(*R*-MPEA)BAPbBr$_4$	850	0.80	[23]
(*R*)-(MBA)$_4$Bi$_2$Br$_{10}$	1064	0.58	[24]

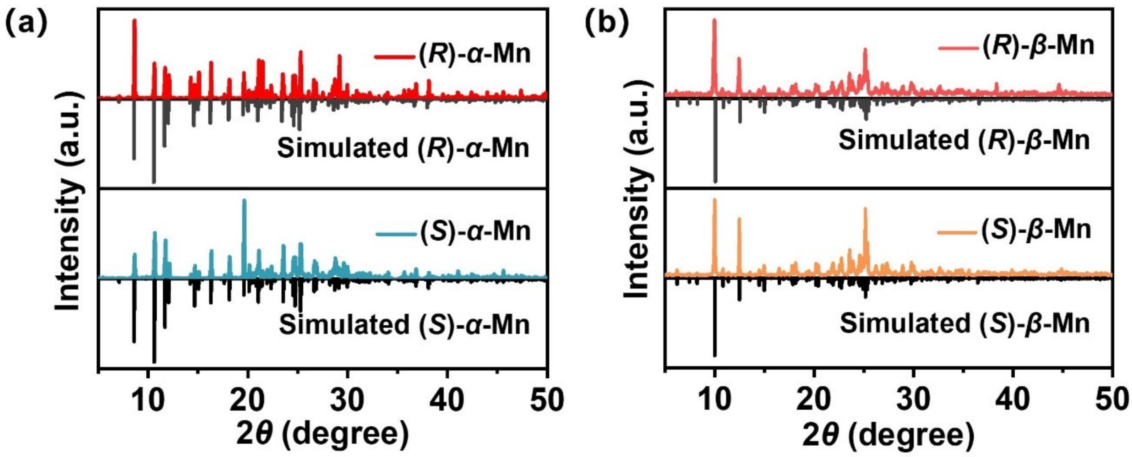

Figure S1 Powder XRD patterns of (*R*)-*α*-Mn, (*S*)-*α*-Mn, (*R*)-*β*-Mn and (*S*)-*β*-Mn.

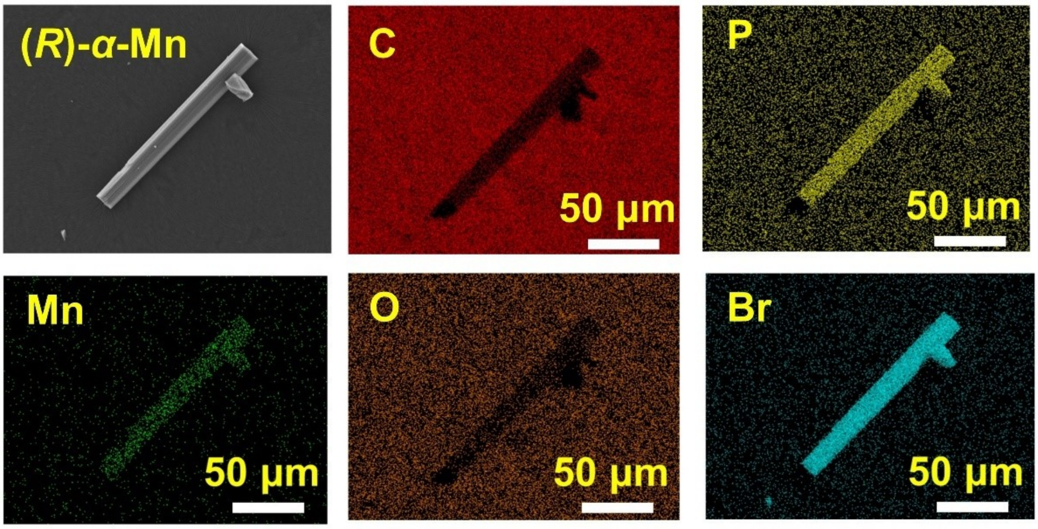

Figure S2 SEM and EDS elemental mapping of (*R*)-*α*-Mn crystals.

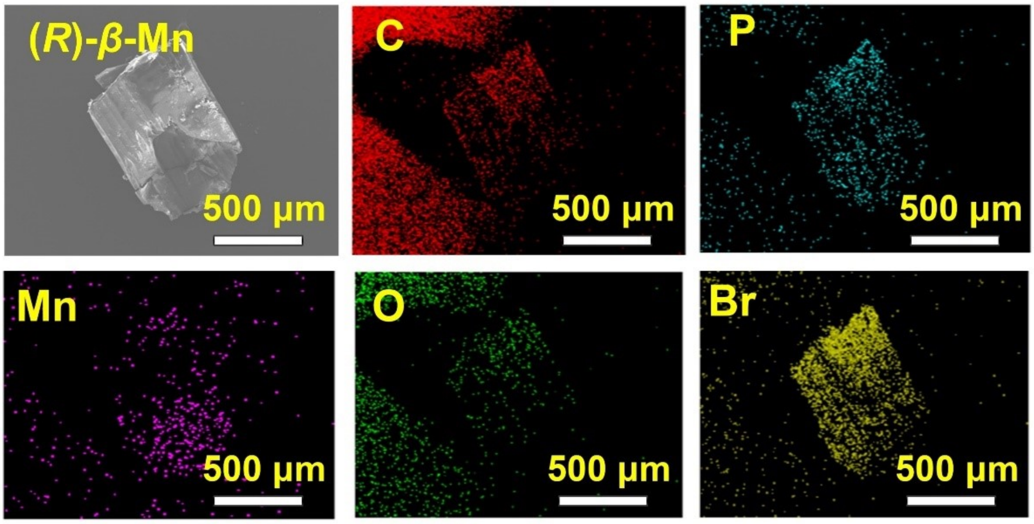

Figure S3 SEM and EDS elemental mapping of (*R*)-*β*-Mn crystals.

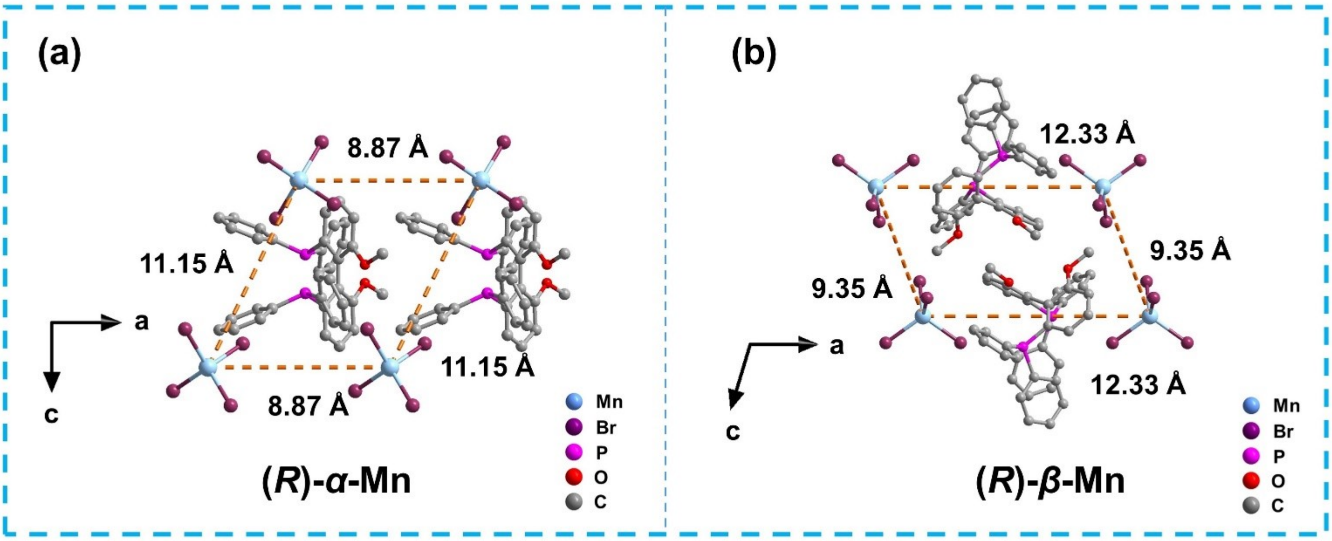

Figure S4 Mn-Mn distance of (*R*)-*α*-Mn and (*R*)-*β*-Mn crystals.

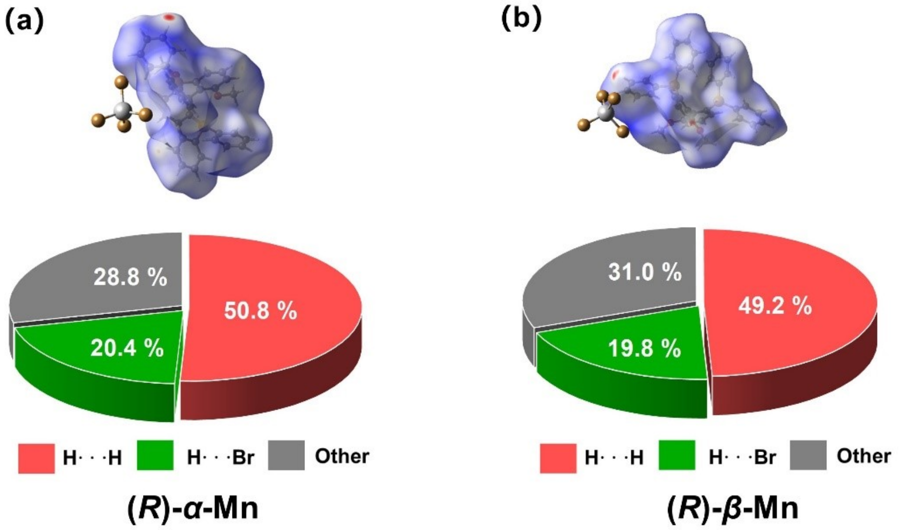

Figure S5 Hirshfeld surface and interaction pie diagram for chiral organics in (*R*)-*α*-Mn (a) and in (*R*)-*β*-Mn (b).

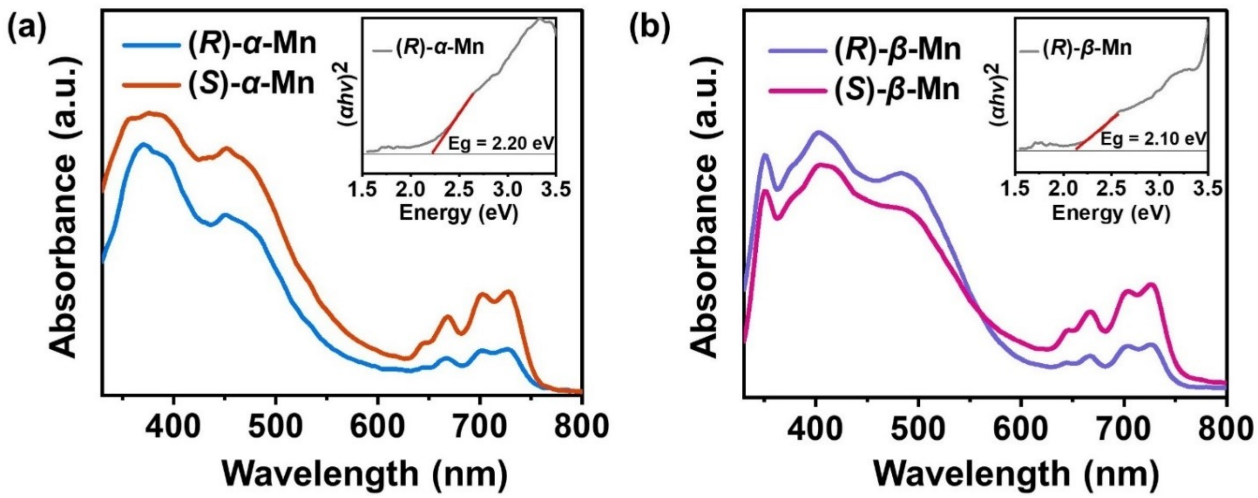

Figure S6 UV-Vis absorbance spectra of (*R*)-*α*-Mn and (*S*)-*α*-Mn (a), (*R*)-*β*-Mn and (*S*)-*β*-Mn (b). The insets show the experimental determination of the band gap (Eg).

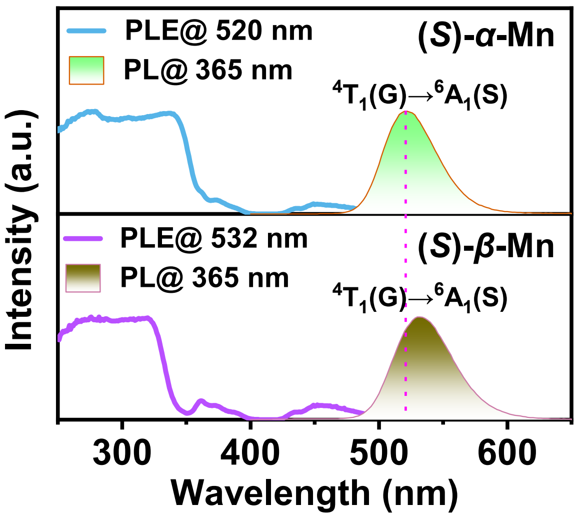

Figure S7 PLE and PL spectra of (*S*)-*α*-Mn and (*S*)-*β*-Mn crystals.

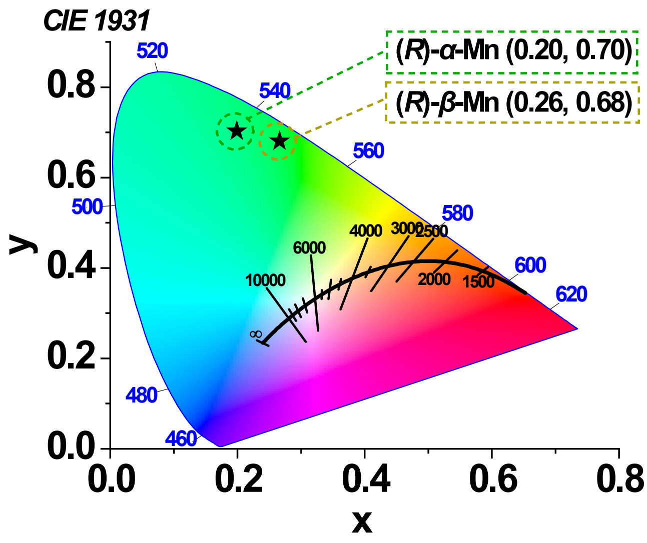

Figure S8 CIE coordinate of (*R*)-*α*-Mn and (*R*)-*β*-Mn crystals.

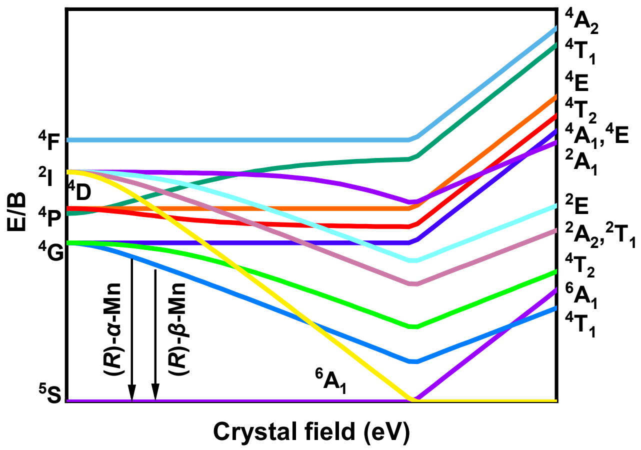

Figure S9 Tanabe-Sugano diagram for the d^5 electron configuration.

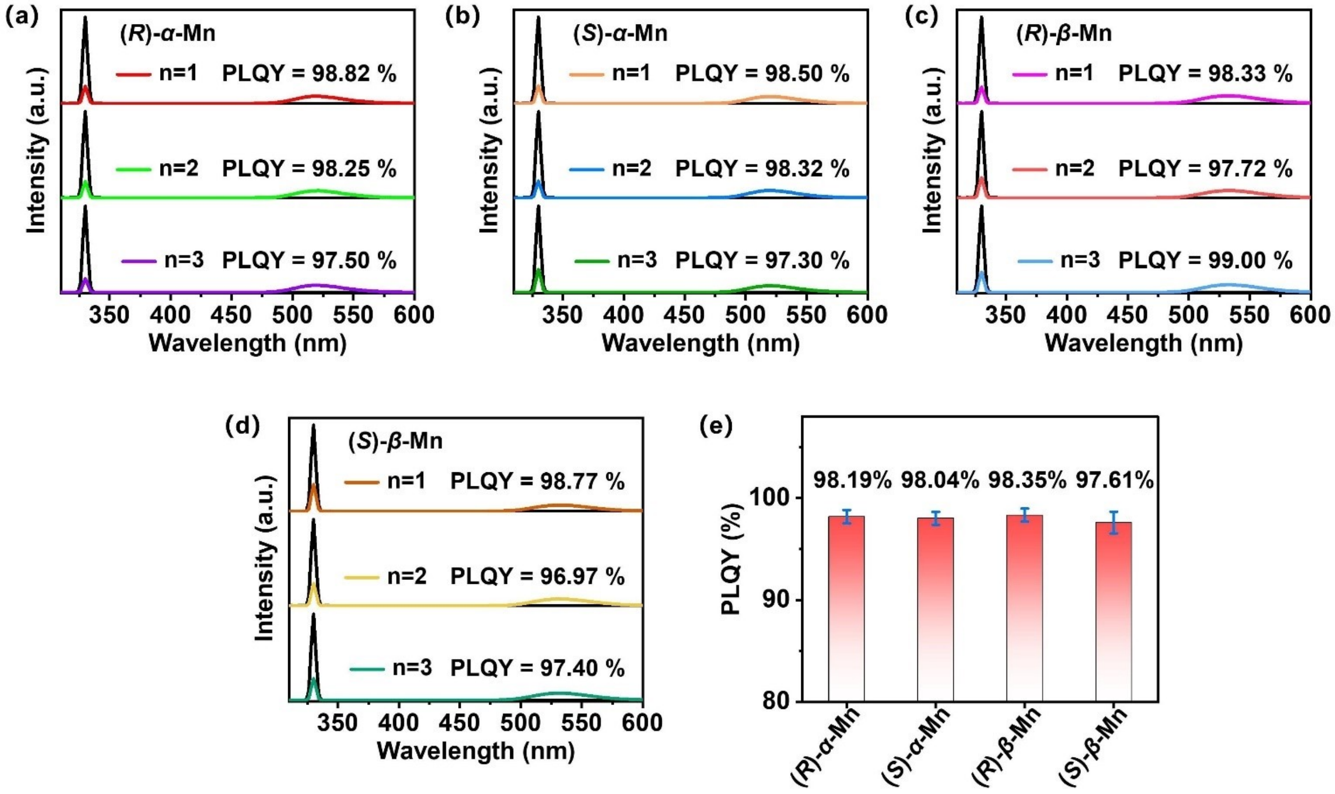

Figure S10 PLQY spectra and error plots from multiple measurements of (*R*)-*α*-Mn, (*S*)-*α*-Mn, (*R*)-*β*-Mn and (*S*)-*β*-Mn crystals (λ_{ex} = 330 nm, n=3).

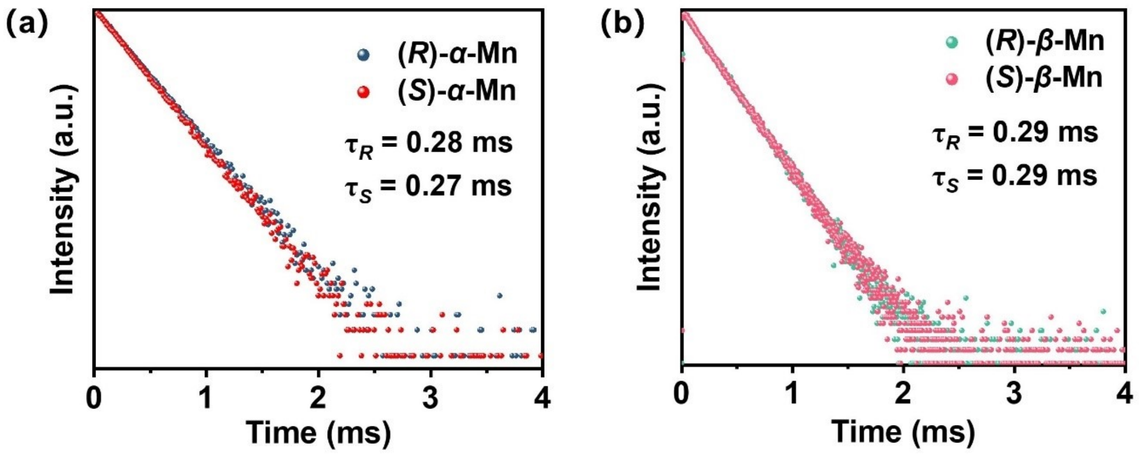

Figure S11 Time-resolved PL profiles of (*R*)-*α*-Mn, (*S*)-*α*-Mn (λ_{em} = 520 nm), (*R*)-*β*-Mn and (*S*)-*β*-Mn (λ_{em} = 532 nm).

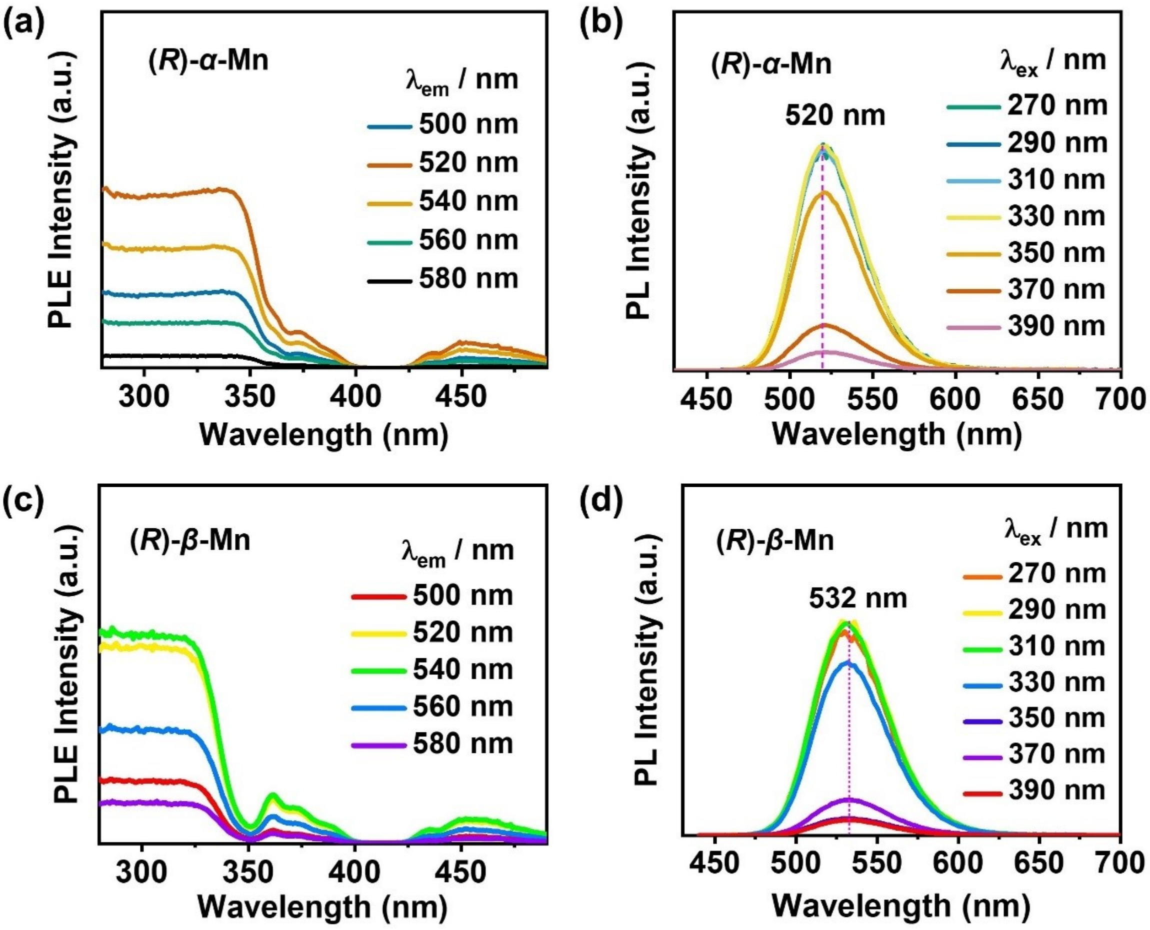

Figure S12 (a) PLE spectra of (*R*)-*α*-Mn crystals by monitoring the emission from Mn^{2+} at different wavelengths. (b) PL spectra of (*R*)-*α*-Mn crystals under excitation at different wavelengths. (c) PLE spectra of (*R*)-*β*-Mn crystals by monitoring the emission from Mn^{2+} at different wavelengths. (d) PL spectra of (*R*)-*β*-Mn crystals under excitation at different wavelengths.

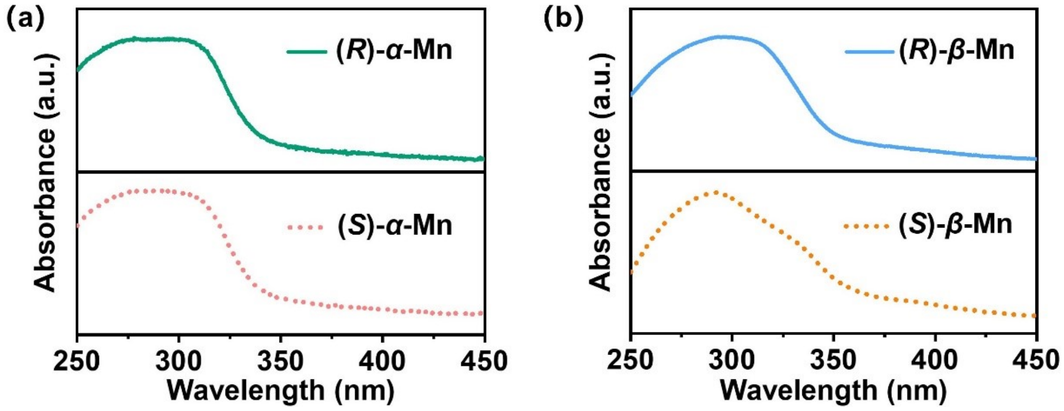

Figure S13 (a-b) Absorbance spectra of (*R*)-*α*-Mn, (*S*)-*α*-Mn (a), (*R*)-*β*-Mn and (*S*)-*β*-Mn (b) samples in KBr pellets.

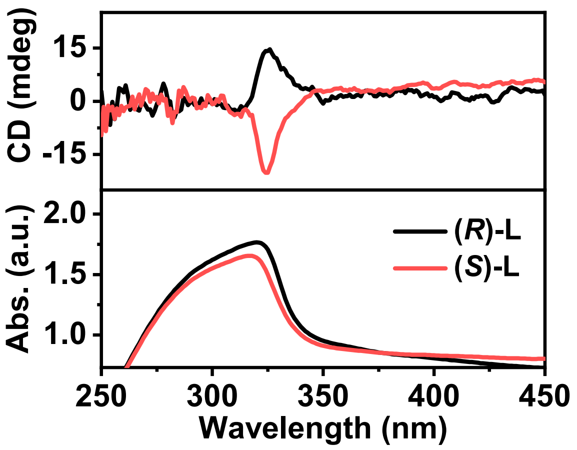

Figure S14 CD spectra of (*R*)-L and (*S*)-L chiral organic salts.

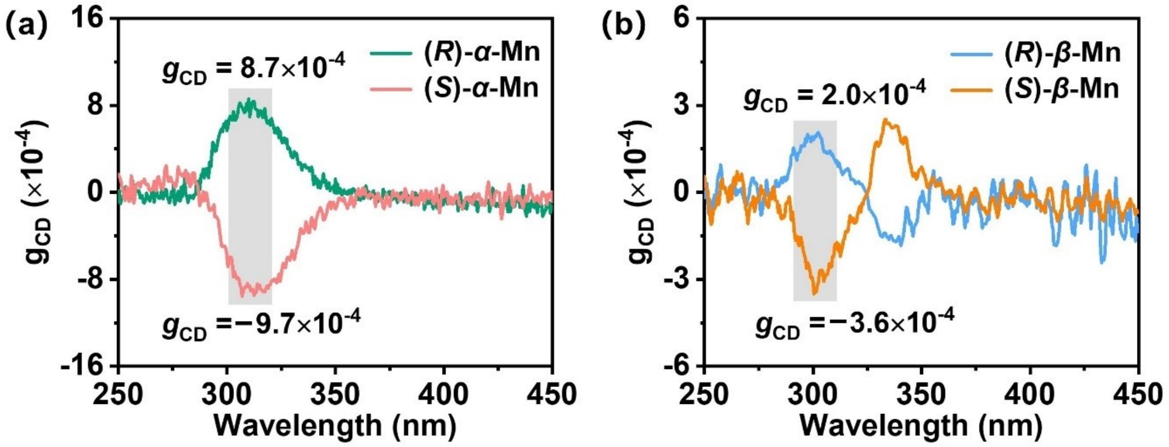

Figure S15 g_{CD} values of (*R*)-*α*-Mn and (*S*)-*α*-Mn (a), (*R*)-*β*-Mn and (*S*)-*β*-Mn (b) samples.

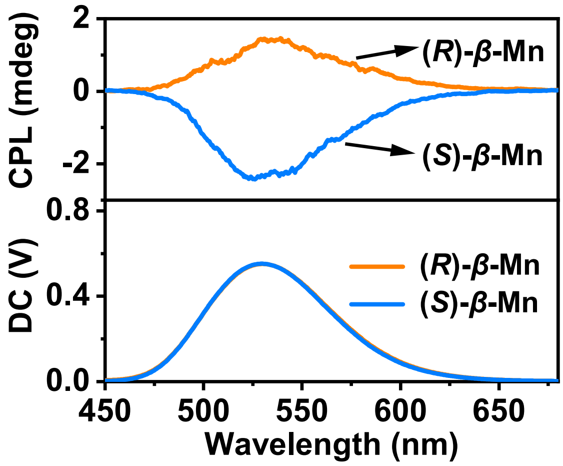

Figure S16 CPL spectra (λ_{ex} = 330 nm) and DC spectra of (*R*)-*β*-Mn and (*S*)-*β*-Mn.

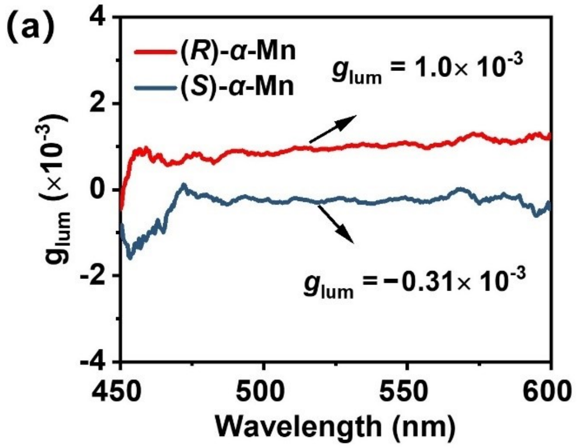

Figure S17 g_{lum} factors associated with emission wavelengths for (*R*)-*α*-Mn and (*S*)-*α*-Mn (a), (*R*)-*β*-Mn and (*S*)-*β*-Mn (b).

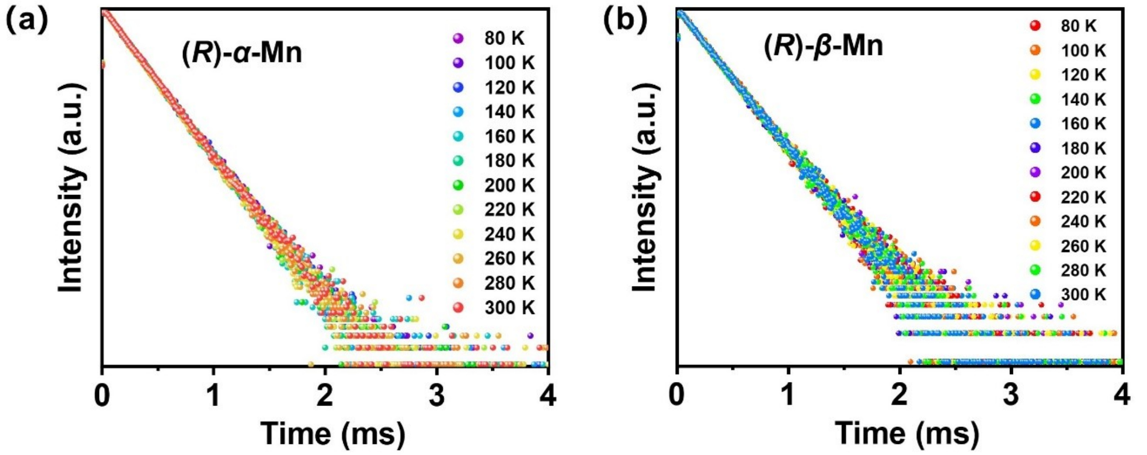

Figure S18 Temperature-dependent PL decay curves of (*R*)-*α*-Mn (λ_{em} = 520 nm) and (*R*)-*β*-Mn (λ_{em} = 532 nm).

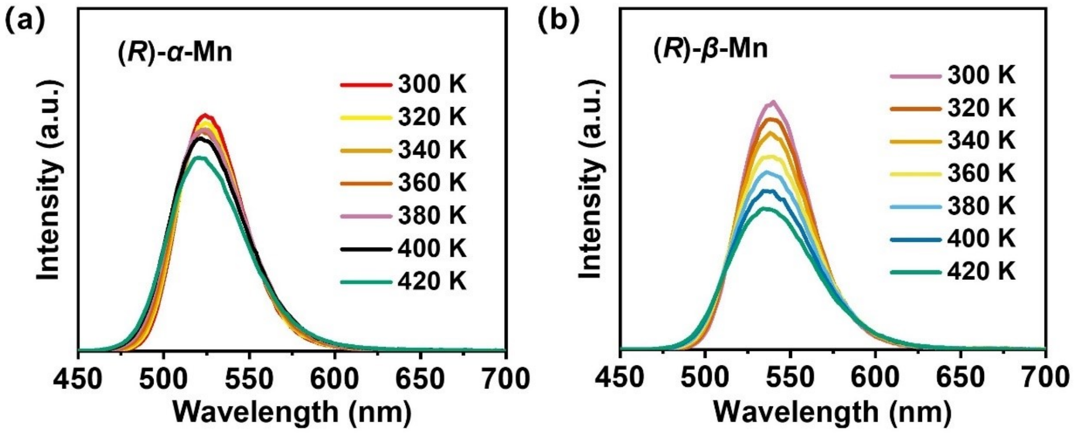

Figure S19 Temperature-dependent PL spectra of (*R*)-*α*-Mn and (*R*)-*β*-Mn crystals recorded from 300 to 420 K.

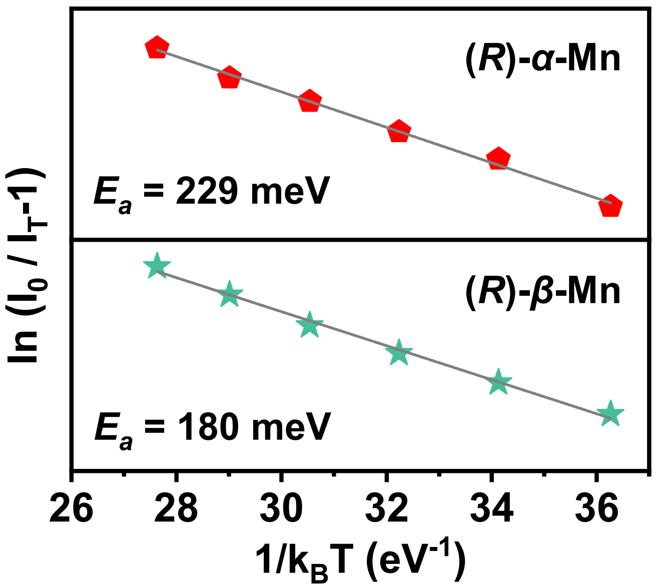

Figure S20 Plot of In(I_0/I_T-1) versus 1/k_BT of the temperature-dependent spectra of (*R*)-*α*-Mn and (*R*)-*β*-Mn.

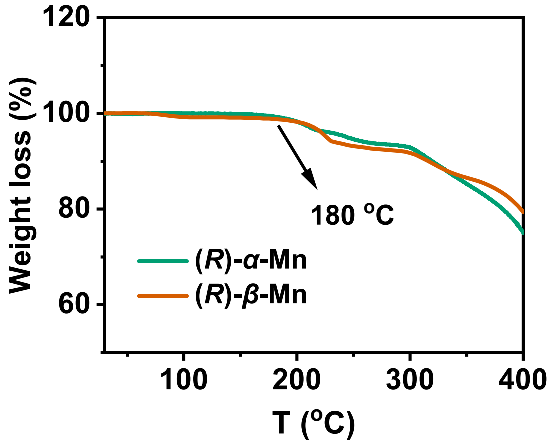

Figure S21 TGA curves for the powder sample of (*R*)-*α*-Mn and (*R*)-*β*-Mn.

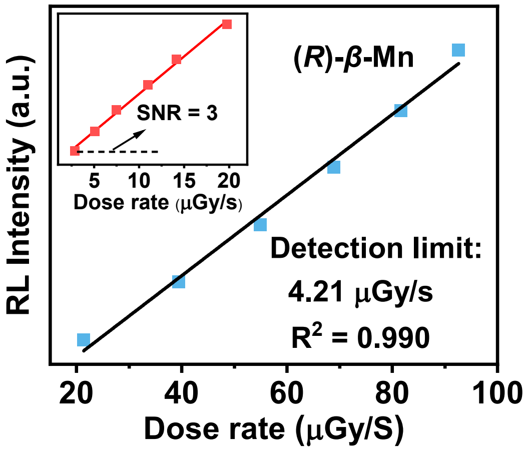

Figure S22 Linear regression analysis of radioluminescence (RL) intensity versus dose rate for (*R*)-*β*-Mn crystals.

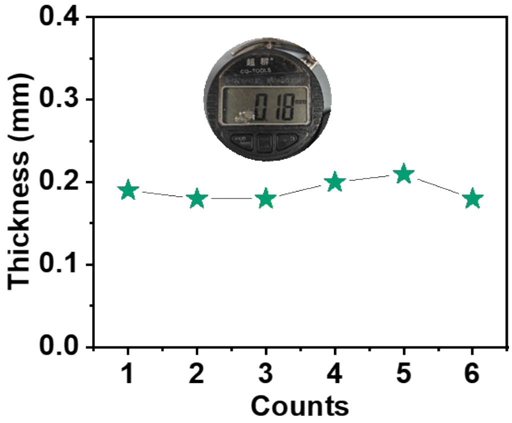

Figure S23 Thickness distribution of the scintillator film.

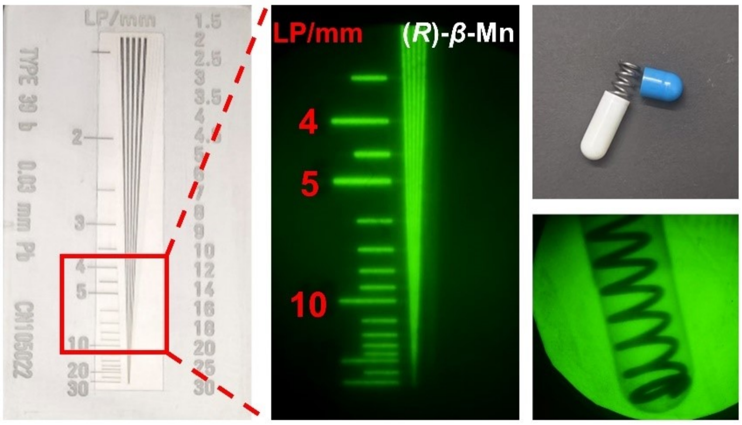

Figure S24 X-ray images, showing scissors with plastic handles, the outline of a metal spring inside the capsule.

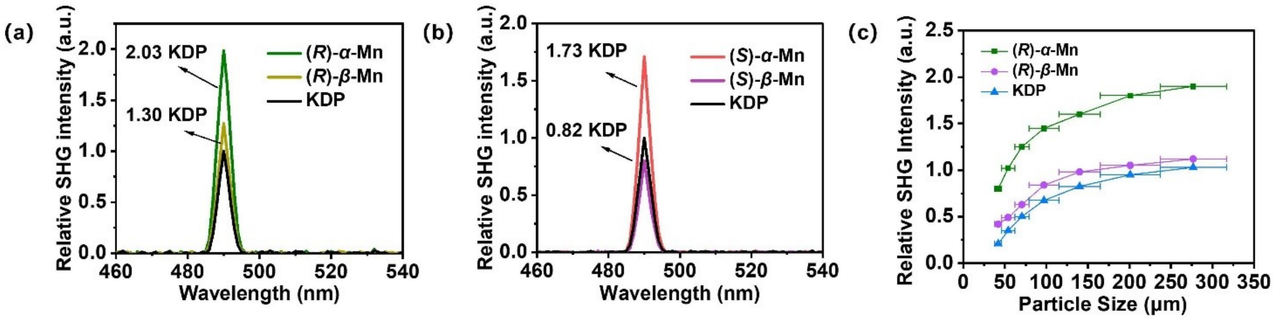

Figure S25 (a) SHG intensity of (*R*)-*α*-Mn and (*R*)-*β*-Mn compared with the standard KDP reference.(b) SHG intensity of (*S*)-*α*-Mn and (*S*)-*β*-Mn compared with the standard KDP reference. (c) Phase matching curves for (*R*)-*α*-Mn, (*R*)-*β*-Mn, and KDP.

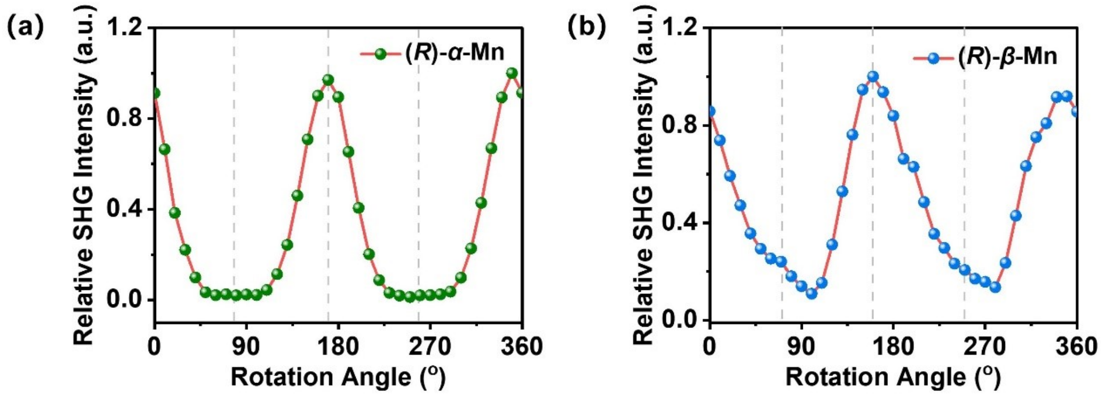

Figure S26 SHG intensity of (R)-α-Mn (a) and (R)-β-Mn (b) samples as a function of the rotation angle of the λ/2 plate.

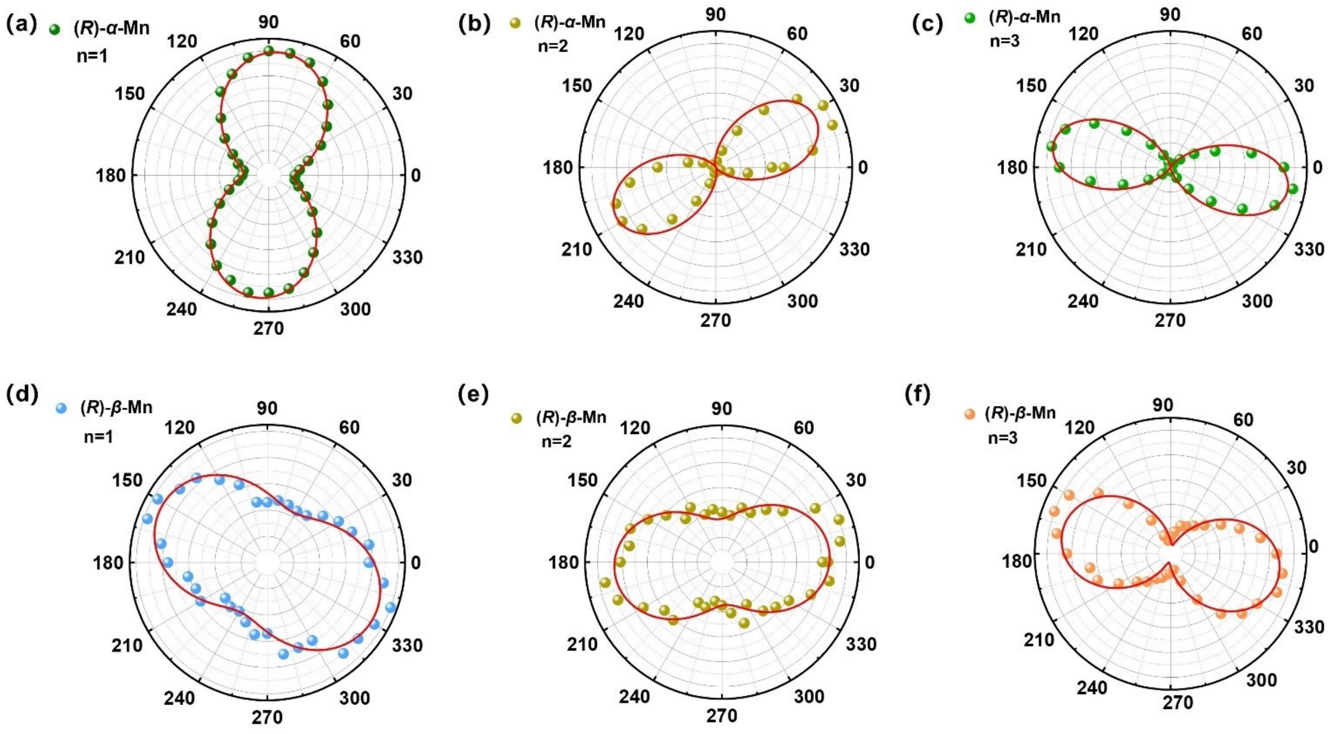

Figure S27 Polarization-dependent SHG of (R)-α-Mn (a-c) and (R)-β-Mn (d-f) under 980 nm pumping (n=3).

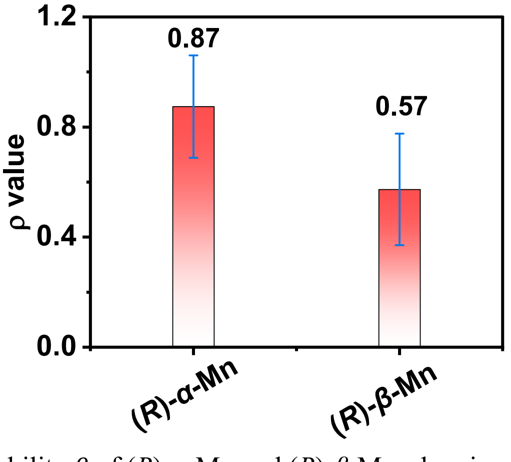

Figure S28 Polarizability ρ of (R)-α-Mn and (R)-β-Mn, showing mean ± s.d. (n=3).

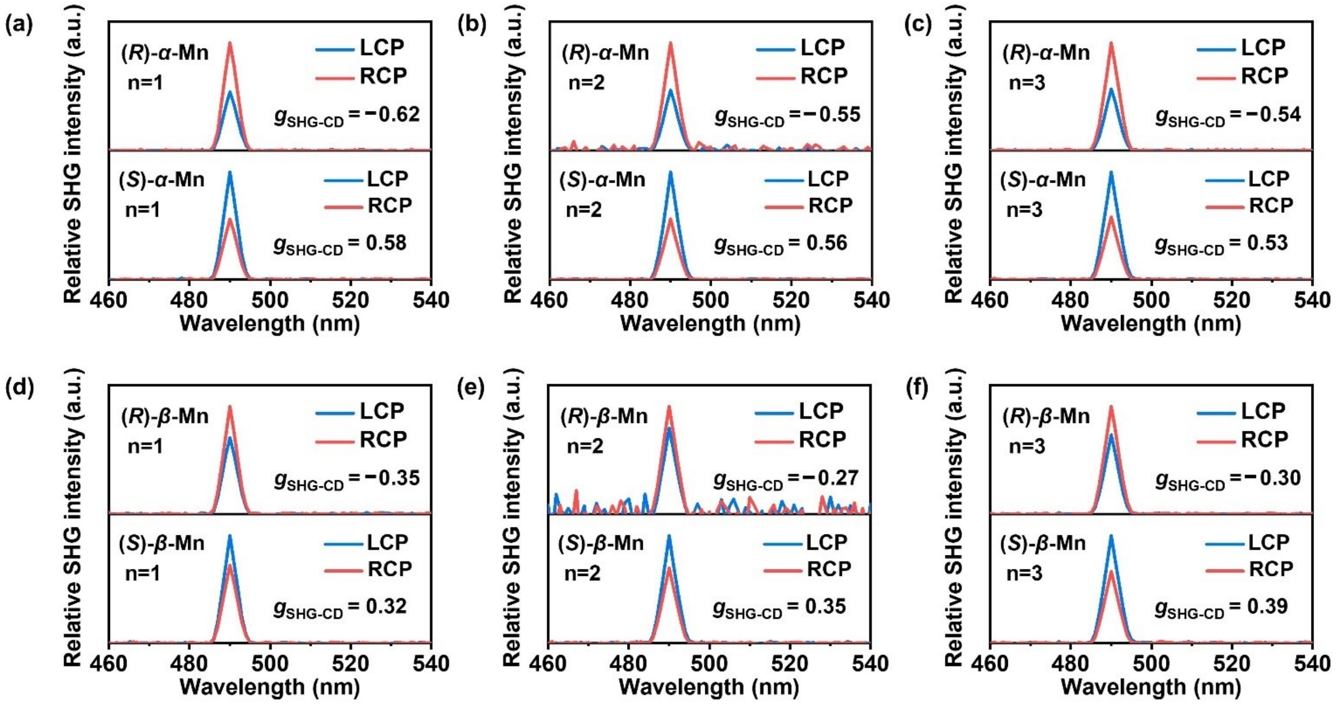

Figure S29 SHG intensity of the (*R*)-*α*-Mn and (*S*)-*α*-Mn samples (a-c), and the (*R*)-*β*-Mn and (*S*)-*β*-Mn samples (d-f), measured under LCP and RCP illumination (n=3).

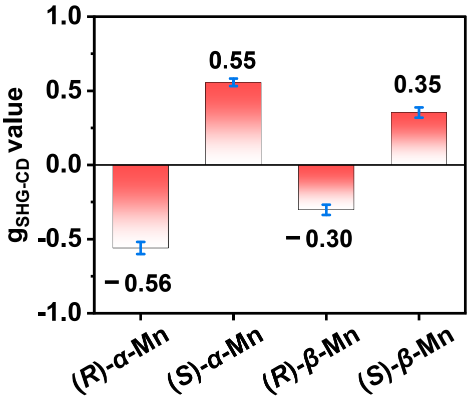

Figure S30 $g_{SHG\text{-}CD}$ values of (*R*)-*α*-Mn, (*S*)-*α*-Mn, (*R*)-*β*-Mn and (*S*)-*β*-Mn.

References

1. Z. Zhang, Z. Wang, H. H. Sung, I. D. Williams, Z. Yu and H. Lu, Revealing the intrinsic chiroptical activity in chiral metal-halide semiconductors, *Journal of the American Chemical Society*, 2022, **144**, 22242-22250.
2. G. Kresse and J. Furthmüller, Efficiency of ab-initio total energy calculations for metals and semiconductors using a plane-wave basis set, *Computational Materials Science*, 1996, **6**, 15-50.
3. G. Kresse and J. Furthmüller, Efficient iterative schemes for ab initio total-energy calculations using a plane-wave basis set, *Physical Review B*, 1996, **54**, 11169.
4. J. P. Perdew, K. Burke and M. Ernzerhof, Generalized gradient approximation made simple, *Physical Review Letters*, 1996, **77**, 3865.
5. S. Grimme, S. Ehrlich and L. Goerigk, Effect of the damping function in dispersion corrected density functional theory, *Journal of Computational Chemistry*, 2011, **32**, 1456-1465.
6. S. Grimme, DFT-D3-A dispersion correction for density functionals, Hartee-Fock and semi-empirical quantum chemical methods DFT-D3, *J Chem Phys*, 2010, **132**, 154104.
7. X. Yu, S. Zhong, Z. Guo, J. Guan, H. Tang, X. He, Y. Chen and S. Pan, Switchable circularly polarized luminescent Mn-based hybrid metal halides, *Journal of Materials Chemistry C*, 2025, **13**, 2190-2197.
8. T. Li, Y. Wang, Y. Liu, G. Liu, L. Meng, Y. Zheng and Y. Dang, Chiral hybrid manganese(II) chloride single crystals for achieving second harmonic generation and moderate circularly polarized luminescence, *Journal of Materials Chemistry C*, 2025, **13**, 8238-8246.
9. L. Zhai, J. Yuan, J. Huang, X. W. Pan, L. Wan, W. Ning and X. M. Ren, Efficient Circularly Polarized Luminescence from Mn-Br Hybrid Perovskite Assembled by Achiral Architectures, *Angewandte Chemie International Edition*, 2025, **64**, e202425543.
10. Z. Z. Huo, Y. Wang, B. Yang, J. Q. Liang, X. F. Hong, Q. An, L. Yuan, H. Ma, J. L. Zuo and Y. X. Zheng, Circularly Polarized Electroluminescence From Chiral Manganese (II) Complexes, *Advanced Optical Materials*, 2025, **13**, 2402684.

11. Y. Wu, S. Wang, Z. Lin, L. Kang, J. Wu, Q. Chen and Z. Lin, Lantern-Shaped Structure Induced by Racemic Ligands in Red-Light-Emitting Metal Halide with Near 100% Quantum Yield and Multiple-Stimulus Response, *Angewandte Chemie International Edition*, 2025, **137**, e202416062.
12. X. He, Y. Zheng, Z. Luo, Y. Wei, Y. Liu, C. Xie, C. Li, D. Peng and Z. Quan, Bright circularly polarized mechanoluminescence from 0D hybrid manganese halides, *Advanced Materials*, 2024, **36**, 2309906.
13. D. Kong, Y. Wu, C. Shi, H. Zeng, L. Xu and Z. Chen, Highly efficient circularly polarized electroluminescence based on chiral manganese(II) complexes, *Chemical Science*, 2024, **15**, 16698-16704.
14. M. Wang, X. Wang, B. Zhang, F. Li, H. Meng, S. Liu and Q. Zhao, Chiral hybrid manganese(II) halide clusters with circularly polarized luminescence for X-ray imaging, *Journal of Materials Chemistry C*, 2023, **11**, 3206-3212.
15. T. Zhang, H. Kang, B. Li, J. Zhou, P. Zhao, T. Zhao, X. Li and S. Jiang, Dynamic cpl switching realized in chiral Mn-based metal halides with reversible thermochromism, *Journal of Materials Chemistry C*, 2023, **11**, 5461-5468.
16. M. P. Davydova, L. Meng, M. I. Rakhmanova, I. Y. Bagryanskaya, V. S. Sulyaeva, H. Meng and A. V. Artem'ev, Highly Emissive Chiral Mn(II) Bromide Hybrids for UV-Pumped Circularly Polarized LEDs and Scintillator Image Applications, *Advanced Optical Materials*, 2023, **11**, 2202811.
17. J. Chen, S. Zhang, X. Pan, R. Li, S. Ye, A. K. Cheetham and L. Mao, Structural origin of enhanced circularly polarized luminescence in hybrid manganese bromides, *Angewandte Chemie International Edition*, 2022, **61**, e202205906.
18. W. Lv, C. Han, L. Zhang, M. Chen, C. He, Y. Tang, J. Wang and R. Chen, Chiral Mn-Zn Hybrid Halides Engineered toward Circularly Polarized Luminescence and X-ray Scintillation Imaging, *The Journal of Physical Chemistry Letters*, 2025, **16**, 8563-8569.
19. B. Wang, C. Wang, Y. Chu, H. Zhang, M. Sun, H. Wang, S. Wang and G. Zhao, Environmental-friendly lead-free chiral Mn-based metal halides with efficient circularly polarized photoluminescence at room temperature, *Journal of Alloys and Compounds*, 2022, **910**, 164892.
20. Z. Guo, J. Li, J. Liang, C. Wang, X. Zhu and T. He, Regulating optical activity and anisotropic second-harmonic generation in zero-dimensional hybrid copper halides, *Nano Letters*, 2022, **22**, 846-852.
21. D. Fu, J. Xin, Y. He, S. Wu, X. Zhang, X. M. Zhang and J. Luo, Chirality-dependent second-order nonlinear optical effect in 1D organic-inorganic hybrid perovskite bulk single crystal, *Angewandte Chemie International Edition*, 2021, **60**, 20021-20026.
22. C. Yuan, X. Li, S. Semin, Y. Feng, T. Rasing and J. Xu, Chiral lead halide perovskite nanowires for second-order nonlinear optics, *Nano Letters*, 2018, **18**, 5411-5417.
23. Z. Guo, J. Li, R. Liu, Y. Yang, C. Wang, X. Zhu and T. He, Spatially correlated chirality in chiral two-dimensional perovskites revealed by second-harmonic-generation circular dichroism microscopy, *Nano Letters*, 2023, **23**, 7434-7441.
24. X. Fu, Z. Zeng, S. Jiao, X. Wang, J. Wang, Y. Jiang, W. Zheng, D. Zhang, Z. Tian and Q. Li, Highly anisotropic second-order nonlinear optical effects in the chiral lead-free perovskite spiral microplates, *Nano Letters*, 2023, **23**, 606-613.
